# Total treatment interval and quality of life of women living with breast cancer in Ethiopia: the mediating role of financial toxicity

**DOI:** 10.1007/s11136-026-04225-9

**Published:** 2026-04-01

**Authors:** Anteneh Ayelign Kibret, Heng Jiang, Edom Seife Woldetsadik, Miliyard Demeke Tafese, Biniyam Tefera Deressa, Chaojie Liu

**Affiliations:** 1 School of Psychology and Public Health, La Trobe University, Bundoora, VIC Australia; 2 Department of Human Anatomy, School of Medicine, College of Medicine and Health Sciences, University of Gondar, Gondar, Ethiopia; 3 School of Population and Global Health, University of Melbourne, Melbourne, VIC Australia; 4 Department of Oncology, College of Health Sciences, Addis Ababa University, Addis Ababa, Ethiopia; 5 Adama Hospital Medical College, Adama, Ethiopia; 6 Jimma Oncology Centre, Jimma University, Jimma, Ethiopia

**Keywords:** Breast cancer, Quality of life, Total treatment interval, Financial toxicity, Ethiopia

## Abstract

**Purpose:**

Delays in breast cancer care are common in low-resource settings and may adversely affect patients’ quality of life (QoL). Prolonged total treatment interval (TTI) can also impose substantial financial hardship. This study aimed to get insights into the potential mechanisms of how prolonged treatment interval impacts QoL through a mediation model and explore the mediating role of financial toxicity (FT) among Ethiopian women with breast cancer.

**Methods:**

A cross-sectional study was conducted among 458 women with histologically confirmed breast cancer receiving treatment at three oncology centers in Ethiopia (Black Lion, Jimma, and Hiwot Fana Hospitals) between July and September 2024. QoL was assessed using the EORTC QLQ-C30, where higher functioning and global health scores indicate better QoL, while higher symptom scores reflect greater symptom burden. Financial toxicity was measured using the COST–FACIT tool, with lower scores indicating greater financial hardship. TTI was calculated as days from symptom recognition to treatment initiation. Mediation and moderated mediation analyses were performed using PROCESS v4.3 for R, controlling for sociodemographic and clinical factors.

**Results:**

Of 456 (99.6%) women with complete data, 71% experienced delayed treatment initiation (> 90 days). The mean (SD) global health status and EORTC QLQ-C30 summary scores were 81.2 (19.9) and 85.8 (15.2), respectively. Longer TTI was significantly associated with poorer QoL, with declines observed in both global health status (*r* = − 0.22, *p* < 0.001) and the QLQ-C30 summary score (*r* = − 0.17, *p* < 0.001). In multivariable models, longer TTI (> 90 days) was associated with lower FT scores, indicating greater financial hardship (β = −2.72, *p* = 0.001). Financial toxicity was positively associated with GHS scores (β = 0.63, *p* < 0.001). The indirect effect of TTI on GHS through financial toxicity was significant (β = −1.70, 95% CI [− 2.94, − 0.66]), while direct and total effects were not. Similar patterns were observed for the EORTC QLQ-C30 summary and functional/symptom domains. Cancer stage did not significantly moderate the indirect pathway.

**Conclusion:**

Prolonged TTI impair quality of life among women with breast cancer in Ethiopia primarily through increased financial hardship rather than direct clinical effects. Interventions aimed at reducing delays and mitigating financial burden may enhance patient well-being and treatment outcomes in low-resource settings.

**Supplementary Information:**

The online version contains supplementary material available at 10.1007/s11136-026-04225-9.

## Background

Breast cancer remains a leading cause of cancer morbidity and mortality among women worldwide [1]. Although breast cancer incidence is higher in high-income countries, about seven in ten breast cancer deaths occur in low- and middle-income countries (LMICs). Ethiopia exemplifies this disparity; breast cancer is the most frequently diagnosed cancer and the leading cause of cancer-related death, accounting for 32% of new cancer cases and 17.6% of cancer deaths [2]. The high mortality in Ethiopia and similar settings is strongly driven by late-stage diagnosis and delays in accessing effective treatment [3–6].

Beyond its impact on survival, breast cancer significantly affects patients’ quality of life (QoL) [7, 8]. Quality of life is a multidimensional concept encompassing physical, psychological, social, and emotional well-being, as perceived by the patient. In oncology, improving patients’ QoL has become a major goal of cancer care, as QoL assessment provides valuable insights into treatment response, tolerance, and prognosis [9, 10]. QoL can be adversely affected following a cancer diagnosis and may further deteriorate during treatment, as the physical, psychological, social, and emotional burdens of the disease and its management intensify [11]. QoL in women with breast cancer is shaped by both the direct effects of the malignancy, such as tumour stage and disease progression, and by burdens imposed by therapies including surgery, systemic treatments, radiotherapy, and hormonal therapy [12]. While oncologic treatments can improve survival and sometimes relieve disease-related symptoms, they frequently lead to a wide range of treatment-related side effects, such as pain, fatigue, lymphedema, altered body image (e.g. following mastectomy or hair loss), which in turn impair daily functioning and psychosocial well-being [13]. With advances in cancer treatment and increasing survival, enhancing QoL of cancer patients has become a central focus of healthcare and better QoL itself has been linked to improved survival [14, 15].

Although many determinants of QoL in breast cancer have been described, the potential influence of timeliness of care remains under-studied. Some studies suggest that longer treatment intervals contribute to disease progression, more intensive treatments, and increased psychological and financial burden, all of which plausibly worsen QoL [16, 17]. The WHO Global Breast Cancer Initiative (GBCI) emphasises that timely diagnosis and treatment completion are critical, not only for improving survival outcomes but also for supporting better patient experience and QoL [18]. A cross-sectional study conducted in Poland found that longer waiting time to initiate treatment was significantly associated with poorer QoL among women with breast cancer [17]. Such prolonged care intervals are common in many LMICs and have profound implications for patient outcomes [19].

Importantly, prolonged total treatment interval (TTI) can expose patients to substantial financial hardship [20, 21]. Extended delays frequently increase the number of diagnostic consultations, repeated imaging or laboratory tests, additional travel to healthcare facilities, longer periods of work absenteeism, and sometimes more advanced disease at presentation that requires costlier and more intensive treatment [22, 23]. These cumulative direct and indirect costs further compromise QoL, suggesting that financial hardship may act as a mediator in the relationship between TTI and QoL. Several studies have consistently shown that financial hardship adversely affects QoL through multiple pathways, including increased psychological distress, impaired treatment adherence, delayed or forgone care, heightened symptom burden, and diminished social functioning [24, 25].

Despite evidence that both delayed care and financial hardship adversely affect QoL, little is known about whether the negative impact of prolonged TTI on QoL operates partly through financial hardship. We therefore hypothesised that longer TTI would be associated with poorer QoL, and that this association would be partly mediated by financial hardship. To date, this mediating relationship has not been adequately examined in Ethiopian patients or more broadly in Africa, where research has largely focused on clinical outcomes rather than patient-reported outcomes. Addressing this gap may clarify how timely care influences survivorship quality and inform strategies to improve the wellbeing and outcomes of women with breast cancer in low-resource settings.

## Method

### Study design, participants and settings

A multicentre cross-sectional study was conducted in three specialised oncology hospitals in Ethiopia: Black Lion Hospital (Addis Ababa), Jimma University Medical Center (Jimma), and Hiwot Fana Comprehensive Specialized Hospital (Harar). Data were collected between July and September 2024. These hospitals provide oncology services to patients from the capital city as well as eastern and southwestern regions of the country.

Participants were recruited using non-probability consecutive sampling, whereby all consecutively eligible women with histologically confirmed breast cancer attending the three hospitals during the study period were invited to participate.

Eligible participants were women aged ≥ 18 years with breast cancer at any stage who had initiated any form of cancer treatment. Women receiving palliative care were excluded because their clinical trajectory and quality-of-life profile differ substantially from patients undergoing curative or life-prolonging treatment. Patients with documented recurrence, severe mental illness, coexisting malignancies, or severe clinical conditions preventing interview participation were also excluded. Information on recurrence status was obtained from medical record review.

### Data collection tools and procedures

Data were collected through an interviewer-administered questionnaire and medical record review. The questionnaire was developed in English using REDCap and tailored to the Ethiopian context and study objectives. Standardised instruments were incorporated, including the EORTC QLQ-C30, which has previously been validated in Ethiopia, and the COST–FACIT financial toxicity scale, which was translated and culturally adapted into Amharic following FACIT translation guidelines as part of our study. The remaining questionnaire items were translated into Amharic to ensure conceptual equivalence and cultural appropriateness. Data collectors administered the Amharic version in interviews and entered responses into REDCap in English. The translated tool was pilot tested to assess clarity, consistency, and question sequence. The questionnaire covered sociodemographic information (age, place of residence, study site, marital status, occupation, educational status, monthly household income, distance from the nearest health facility, health insurance status, and family size), clinical characteristics (presence of medically confirmed comorbidities, treatment stage, cancer stage, treatment modality, hospitalization history, and body mass index), key dates of the care pathway including symptom recognition, diagnosis, and treatment initiation, financial toxicity measured by the COST–FACIT tool [26], and QoL measured by the EORTC QLQ-C30 [27]. Demographic and clinical variables measured in this study were identified through a review of relevant literature as they are key determinants of QoL. For instance, factors such as age, education, income, cancer stage, and comorbidity have been shown to be significant predictors of QoL among women with breast cancer in previous studies [28–30]. Monthly household income was self-reported by participants and based on the distribution of responses, income was categorized into tertiles: Low (Tertile 1): ≤ 2,100 Ethiopian Birr (ETB), Medium (Tertile 2): 2,101–3,254 ETB, and High (Tertile 3): 3,255–15,000 ETB. Cancer stage at diagnosis was categorized as early stage (stages I–II) or late/advanced stage (stages III–IV), based on the American Joint Committee on Cancer (AJCC) TNM classification system [31]. Comorbidity refers to the presence of at least one extra chronic illness along with a chronic disease of interest [32]. Chronic illness are defined as irreversible conditions with no complete recovery or as long-lasting illnesses that persist over time [33]. Treatment modality was grouped by intensity into single-modality (surgery, chemotherapy, or radiotherapy only), dual-modality (any two of these treatments), and triple-modality therapy (all three). Days since treatment initiation was categorized according to time elapsed since treatment initiation to reflect key milestones along the breast cancer care continuum, conceptually informed by the phases described in the NCCN Survivorship Guidelines: Based on key clinical milestones, participants were categorised into four stages ≤ 14 days (just started), 15–180 days (early–mid treatment), 181–365 days (late treatment), and > 365 days (post-treatment).

Three trained female medical doctors, who were not involved in patient care, conducted face-to-face interviews with participants, and entered data into the Redcap. Women diagnosed with primary breast cancer who had initiated any form of treatment were approached in person by the data collectors during routine clinic visits or hospital stays. After confirming eligibility, the purpose of the study was explained, and written informed consent was obtained prior to the interview. To ensure data quality, the first author provided daily supervision and conducted weekly site visits during the data collection period.

A total of 458 women consented and initiated the survey. Two participants discontinued participation due to fatigue during the interview and were therefore excluded. The remaining 456 participants completed the survey and constituted the final analytic sample included in the analysis.

### Measures

#### Quality of life (QoL)

The European Organization for Research and Treatment of Cancer (EORTC) developed the EORTC QLQ-C30 in 1987 to provide a standardized instrument for assessing QoL in cancer patients [27]. The questionnaire was proved to be valid and reliable to assess QoL in cancer patients in Ethiopia [34].

The EORTC QLQ-C30 measures three broad domains over the past one week: functional, global health status (GHS), and symptom domains. The functional domain contains fifteen items measuring five functioning: physical (five items), role (two items), emotional (four items), cognitive (two items), and social functioning (two items). The global health status domain consists of two items assessing overall health. The symptom domain comprises twelve items measuring fatigue (three items), pain (two items), nausea/vomiting (two items), dyspnoea (one item), insomnia (one item), appetite loss (one item), constipation (one item), and diarrhoea (one item). There is an additional item in the EORTC QLQ-C30 measuring financial difficulties.

Each item is rated on a four-point Likert scale (from 1 “not at all” to 4 “very much”), except for the two GHS items, which use a seven-point scale (from 1 “very poor” to 7 “excellent”).

The item scoring procedure for the EORTC QLQ-C30 was managed according to the EORTC QLQ-C30 scoring manual [35]. For each domain, the raw score (RS) was calculated as the mean of the domain’s item responses:

$$ {\mathrm{Raw}}\;{\mathrm{score}}:RS = \frac{{\left\{ {I1 + I2 + \cdots + In} \right\}}}{n} $$

where n stands for the number of items that form the domain. Domain scores were linearly transformed to a 0–100 scale using the standard EORTC scoring algorithm, ensuring comparability across different domains [36]. For the functioning domain, this formula inverts the raw average because a lower raw score signifies better functioning. By subtracting from 1, the transformation ensures that a higher transformed score indicates better functioning.

$$ {\mathrm{Functional}}\;{\mathrm{domain}}:{\mathrm{S}} = \left\{ {1 - \frac{{RS - 1}}{{range}}{\text{ }}} \right\} \times 100 $$

For the symptom domain, a higher raw score means worse symptoms. A direct linear formula is used so that a higher transformed score reflects a higher level of symptomatology or problems.

$$ {\mathrm{Symptom}}\;{\mathrm{domain}}:{\mathrm{S}} = \left( {\frac{{RS - 1}}{{ range}}} \right) \times 100 $$

The GHS domain is transformed in a similar fashion to the symptom scales. Here, however, a higher raw value means better QoL (since 7 = “Excellent”). No inversion is needed in the formula because the raw coding already aligns with positive outcome.

$$ {\mathrm{Global}}\;{\mathrm{health}}\;{\mathrm{status}}\;{\mathrm{domain}}:{\mathrm{S}} = \left( {\frac{{RS - 1}}{{range}}} \right) \times 100 $$

In addition to the domain-specific scores, an EORTC QLQ-C30 summary score was calculated following the approach proposed by Giesinger et al. [37], who developed and validated this composite indicator to provide a single measure of overall cancer-related QoL. The EORTC QLQ-C30 summary score was calculated as the mean of five functional and eight symptom scores that together represent 27 of the 30 items in the questionnaire, excluding the GHS and financial difficulty items. The symptom scores were reverse scored so that higher values reflected better QoL, consistent with the direction of the functional scores.

The Global Health Status (GHS) score was specified a priori as the primary outcome, reflecting overall quality of life. The summary score and selected functional and symptom domains were analysed as secondary outcomes to explore domain-specific associations.

Using both GHS and the summary score allowed us to capture complementary dimensions of quality of life, encompassing both subjective global well-being and functional and symptom-related aspects of daily living.

#### Financial toxicity (FT)

Financial toxicity was assessed by the Comprehensive Score for financial Toxicity (COST) Measure (Version 2), which was developed by de Souza et al. [26]. The COST includes 12 items and is scored on a 5-point Likert scale (0 = not at all, 4 = very much). The total score ranging from 0 to 44, with higher score indicating lower financial toxicity or hardship.

#### Total treatment interval (TTI)

The total treatment interval (TTI) was defined as the number of days from symptom recognition to the start of breast cancer treatment, based on patient reports verified with medical records. Following the WHO Global Breast Cancer Initiative framework, a 90-day threshold (≤ 90 days = timely; >90 days = delayed) was used, reflecting 60 days for diagnosis and 30 days for treatment initiation [18].

### Statistical analysis

Descriptive statistics were used to summarise socio-demographic, clinical and services characteristics of study participants, as well as the three main domains of the EORTC QLQ-C30 (functioning, symptoms, GHS) and its QoL summed score. Group comparisons of the EORTC QLQ-C30 summed scores and GHS scores were performed using Kruskal–Wallis or Wilcoxon rank-sum tests, as appropriate, because EORTC QLQ-C30 scores and treatment interval variables were not normally distributed based on visual inspection of histograms and Shapiro–Wilk tests. Pearson’s correlation coefficients were calculated to examine the associations of TTI and FT with the EORTC QLQ-C30 summed scores and GHS scores.

Missing data were minimal; BMI category data were missing for two participants (0.4%), and missingness for other variables was negligible. Analyses were therefore conducted using complete-case data.

To examine whether FT mediated the association between TTI and the EORTC QLQ-C30 summed scores and GHS scores, we applied the PROCESS macro v4.3 for R [38] in linear regression, specifying Model 4 with TTI as the predictor, FT (COST-FACIT score) as the mediator, and QoL (measured by both the EORTC QLQ-C30 summed scores and GHS scores) as outcomes. We also examined cancer stage at diagnosis as a potential moderator of the indirect pathway between TTI and QoL using PROCESS Model 59. The modelling analyses controlled for relevant socio-demographic and clinical covariates. Indirect effects were estimated using ordinary least-squares regression and bias-corrected bootstrapped 95% confidence intervals (5,000 resamples).

We further performed sensitivity tests in the linear regression modelling by replacing the EORTC QLQ-C30 summed scores and GHS scores with a functioning composite (mean of the five-functioning sub-domains) and a symptom composite (mean of the eight-symptom sub-domains, reversed so that higher scores indicate better QoL) score. We also tested the regression models using continuous TTI instead of the binary measure.

Multicollinearity was assessed using variance inflation factors (VIF), with no concerns detected (all VIF < 2). All statistical analyses were performed using R version 4.4.3 (R Core Team, 2024).

### Hypotheses

#### H1 (Mediation)

**Fig. 1:**
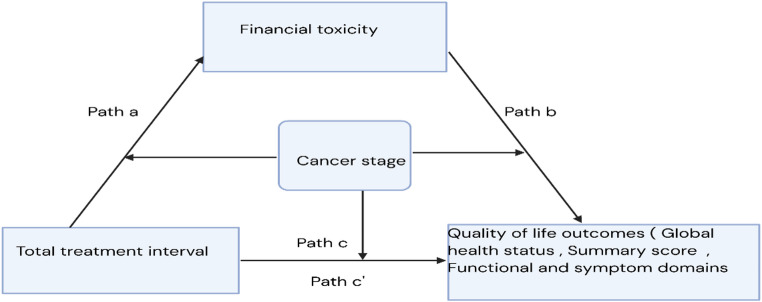
Conceptual model: This figure illustrates the hypothesized moderated mediation model. Total treatment interval (TTI) influences quality of life (QoL) directly and indirectly via financial toxicity (FT). Cancer stage is modelled as a moderator of the a-path (TTI → FT), b-path (FT → QoL), and c′-path (TTI → QoL)

Longer TTI is associated with lower FT score (path a). Lower FT score, in turn, is associated with lower QoL (path b). Thus, FT is hypothesized to mediate the association between TTI and QoL (indirect effect = a × b) (Fig. 1).

#### H2 (moderated mediation)

Cancer stage at diagnosis is hypothesized to moderate the mediation model, such that the strength of associations differs by stage. Specifically, stage of cancer may moderate: a: TTI → FT: b: FT → QoL, and c′: TTI → QoL (direct).

Potential sources of bias were addressed through consecutive recruitment of all eligible participants during the study period to minimise selection bias; use of validated instruments; and standardised interviewer-administered questionnaires with training and supervision of data collectors to reduce interviewer bias. To minimise recall bias related to symptom onset and first healthcare visit dates, participants were asked to anchor events to familiar calendar markers (e.g., religious or national holidays, New Year celebrations, or school holidays), and responses were cross-checked with medical records when available. When exact dates could not be recalled, standardised rules were applied (15th of the month if only the month was remembered, 5th if early in the month, and 25th if late in the month). In addition, multivariable analyses adjusted for key socio-demographic and clinical confounders.

## Result

A total of 458 women consented and initiated the survey. Two participants discontinued participation due to fatigue during the interview and were therefore not included in the final analysis. The remaining 456 participants completed the survey and constituted the final analytic sample. Of the 456 study participants for QoL analyses, most were aged 40–59 years (51.8%), urban residents (73.8%), and recruited mainly from Black Lion Hospital (55%). The majority were married (62%), unemployed (51.5%), had primary/secondary schooling (45.4%), TTI > 90 days (71%), stage III disease and IV (58.8%), post-treatment phase (55%), and BMI in the normal range (73.5%).

The mean (SD) GHS and EORTC QLQ-C30 summed score were 81.2 (19.9) and 85.8 (15.2), respectively. Women aged 40–59 years reported the highest QoL (GHS 83.4; summed QoL 87.4), whereas those ≥ 60 years scored lowest (GHS 76.1; summed QoL 80.7; *p* = 0.016 and 0.003, respectively). Urban residents had markedly better QoL than rural residents (GHS 84.9 vs. 70.4; summed QoL 87.6 vs. 80.8; *p* < 0.001). Participants with higher education and higher household income also had significantly better scores than those with no schooling or lowest income tertile (both *p* < 0.001). QoL varied substantially by study site, being highest at Black Lion and lowest at Hiwot Fana (both scales *p* < 0.001). Shorter total treatment interval (≤ 90 days) was linked to better QoL (GHS 87.0 vs. 78.8; summed QoL 89.7 vs. 84.2; *p* < 0.001). Stage IV patients had the lowest scores (GHS 65.9; summed QoL 74.7; *p* < 0.001). QoL was sharply lower among women with ≥ 4 hospitalizations in the past year (GHS 56.8; summed QoL 64.6; *p* < 0.001) (Table 1). Patterns were largely consistent for both GHS and the EORTC QLQ-C30 summed scores.

**Table 1:** Global health status and QLQ-C30 summed scores by patient characteristics

Characteristic	*n* (%)	GHS	*p*-value	EORTC QLQ-C30 summary score	*p*-value
Mean (SD)		81.2 (19.9)		85.8 (15.2)	
Age (Years)			0.016		0.003
< 40	168 (36.7)	79.7 (19.6)		85.2 (14.2)	
40–59	237 (51.8)	83.4 (19.3)		87.4 (14.8)	
≥ 60	53 (11.5)	76.1 (22.3)		80.7 (18.7)	
Residence			< 0.001		< 0.001
Urban	338 (73.8)	84.9 (18.4)		87.6 (14.0)	
Rural	118 (25.8)	70.4 (20.3)		80.8 (17.4)	
Study site			< 0.001		< 0.001
Black Lion	252 (55.0)	86.1 (18.8)		87.2 (14.4)	
Hiwot Fana	65 (14.2)	63.6 (20.3)		72.6 (18.8)	
Jimma	141 (30.8)	80.6 (17.0)		89.3 (11.1)	
Marital status			0.025		0.055
Married	284 (62.0)	82.9 (19.0)		86.9 (14.9)	
Unmarried	174 (38.0)	78.4 (21.2)		84.1 (15.6)	
Occupational status			< 0.001		< 0.001
Employed	222 (48.5)	85.3 (17.8)		88.4 (13.1)	
Unemployed	236 (51.5)	77.3 (21.1)		83.3 (16.6)	
Educational attainment			< 0.001		< 0.001
No formal education	152 (33.2)	73.7 (22.0)		80.8 (17.8)	
Primary & secondary	208 (45.4)	84.0 (18.0)		87.7 (13.5)	
Higher education	98 (21.4)	86.9 (16.9)		89.7 (11.8)	
Monthly household income (ETB) tertiles			< 0.001		< 0.001
≤ 2,100 (T1)	177 (38.7)	76.9 (21.3)		81.9 (16.4)	
2,101–3,254 (T2)	129 (28.2)	81.4 (19.1)		86.5 (14.7)	
3,255–15,000 (T3)	152 (33.2)	86.0 (18.0)		89.8 (12.9)	
Distance to the nearest health facility (km)			0.656		0.724
< 5	350 (76.4)	81.4 (19.9)		85.6 (15.4)	
≥ 5	108 (23.6)	80.4 (20.2)		86.6 (14.7)	
Health insurance			0.649		0.738
Yes	367 (80.1)	81.2 (20.3)		85.9 (15.3)	
No	91 (19.9)	81.0 (18.6)		85.3 (14.8)	
Total Treatment Interval (TTI)			< 0.001		< 0.001
≤ 90 days	133 (29.0)	87.0 (18.6)		89.7 (14.7)	
> 90 days	325 (71.0)	78.8 (20.0)		84.2 (15.1)	
Family size			0.016		0.407
< 2	96 (21.0)	82.5 (20.4)		86.5 (14.8)	
2–5	297 (64.9)	82.2 (19.4)		86.5 (14.5)	
> 6	65 (14.2)	74.9 (20.8)		81.7 (18.2)	
Stage at diagnosis			< 0.001		< 0.001
Stage I	17 (3.7)	79.9 (19.3)		88.7 (8.9)	
Stage II	172 (37.6)	85.1 (18.7)		88.3 (14.3)	
Stage III	212 (46.3)	82.3 (19.4)		86.6 (15.0)	
Stage IV	57 (12.5)	65.9 (19.2)		74.7 (15.7)	
Days since treatment initiation			< 0.001		< 0.001
Just started	14 (3.1)	58.3 (18.8)		67.8 (23.5)	
Early/Mid-treatment	105 (23.0)	69.0 (18.6)		78.8 (16.6)	
Late treatment	86 (18.9)	78.1 (20.6)		84.0 (16.3)	
Post-treatment	251 (55.0)	88.6 (16.4)		90.4 (11.2)	
Medically confirmed chronic illness (comorbidities)			0.319		0.668
No	371 (81.0)	81.1 (19.4)		86.0 (15.1)	
Yes	87 (19.0)	81.7 (22.2)		84.8 (15.8)	
Treatment modality			< 0.001		< 0.001
Single modality	75 (16.4)	67.2 (18.8)		75.7 (18.0)	
Two modalities	301 (65.7)	82.1 (19.6)		86.5 (14.4)	
All three modalities	82 (17.9)	90.9 (14.6)		92.5 (9.5)	
Hospitalizations (past year)			< 0.001		< 0.001
0–3	442 (96.5)	82.1 (19.1)		86.6 (14.3)	
4–7	16 (3.5)	56.8 (27.3)		64.6 (21.9)	
BMI (kg/m²)			< 0.001		< 0.001
Underweight (< 18.5)	25 (5.5)	63.7 (20.7)		74.6 (21.3)	
Normal (18.5–24.9)	335 (73.5)	81.4 (19.8)		86.5 (14.2)	
Overweight (25–29.9)	72 (15.8)	81.6 (19.3)		84.1 (17.2)	
Obese (30–39.9)	24 (5.3)	95.1 (9.2)		93.5 (8.07)	
Traditional healing			0.008		< 0.001
Yes	85 (18.6)	75.1 (22.3)		78.8 (18.4)	
No	373 (81.4)	82.5 (19.1)		87.4 (13.9)	
Financial source of out-of-pocket money (OOP)			0.832		0.967
Self-financed	167 (36.5)	81.2 (19.5)		85.5 (15.5)	
Borrowed/Assisted	291 (63.5)	81.1 (20.2)		86.0 (15.0)	
Avoided care due to cost			0.002		0.013
No	441 (96.3)	81.8 (19.4)		86.2 (14.8)	
Yes	17 (3.7%)	64.2 (25.3)		76.0 (20.2)	

GHS, global health status, c30summary = EORTC QLQ-C30 Summary score; n (%) based on total sample (*N* = 458); mean (SD) of QoL scales based on participants with valid QoL data (*N* = 456).

### Health-related quality of life according to EORTC QLQ-C30

The mean scores for all functioning sub-domains of the EORTC QLQ-C30 were generally high (≥ 80), except for the emotional functioning: cognitive functioning (CF) 88.3 (SD = 21.0), physical functioning (PF) 82.0 (SD = 17.9), role functioning (RF) 81.2 (SD = 24.2), and social functioning (SF) 80.2 (SD = 23.9), and emotional functioning (EF) 77.0 (SD = 19.7). Functioning varied across sociodemographic and clinical groups. A shorter TTI (≤ 90 days) was associated with higher physical, role, and social functioning scores (all *p* < 0.001). Patients with comorbidities showed lower physical and social functioning. Marked site differences were observed, with Hiwot Fana reporting lower functioning across domains (all *p* < 0.001). Advanced stage was linked to lower functioning (e.g., social functioning decreased from 86.3 ± 15.9 at Stage I to 63.5 ± 27.0 at Stage IV; *p* < 0.001). Functioning scores increased with treatment progress. Patients who had just started treatment had the lowest PF (61.9 ± 23.0), while post-treatment patients had the highest scores (PF = 87.4 ± 13.3; RF = 90.1 ± 16.4; SF = 87.5 ± 17.5).

The symptoms with the highest means were fatigue and pain, 26.1 (SD = 21.4) and 19.9 (SD = 24.8), respectively. Financial difficulties were reported as a prominent problem, with a mean of 43.9 (SD = 33.7). Across symptom domains of the EORTC QLQ-C30, notable sociodemographic and clinical differences were observed, following patterns similar to the functioning domains. Women with longer TTI (> 90 days) reported higher levels of fatigue, pain, dyspnoea, insomnia, appetite loss, and financial difficulties (all *p* < 0.05). Rural residents consistently showed higher symptom burdens across multiple domains (all *p* < 0.001). Symptom severity increased with advancing stage, particularly for fatigue, pain, and financial difficulties (all *p* < 0.001). Treatment status also showed a clear gradient, with the highest symptom levels among patients who had just started treatment and the lowest among those who had completed treatment (all *p* < 0.001). Lower education, lower income, and comorbidities were associated with higher symptom scores in several domains (Supplementary Table S1).

### Correlations between EORTC QLQ-C30 scales and total treatment interval

**Fig. 2:**
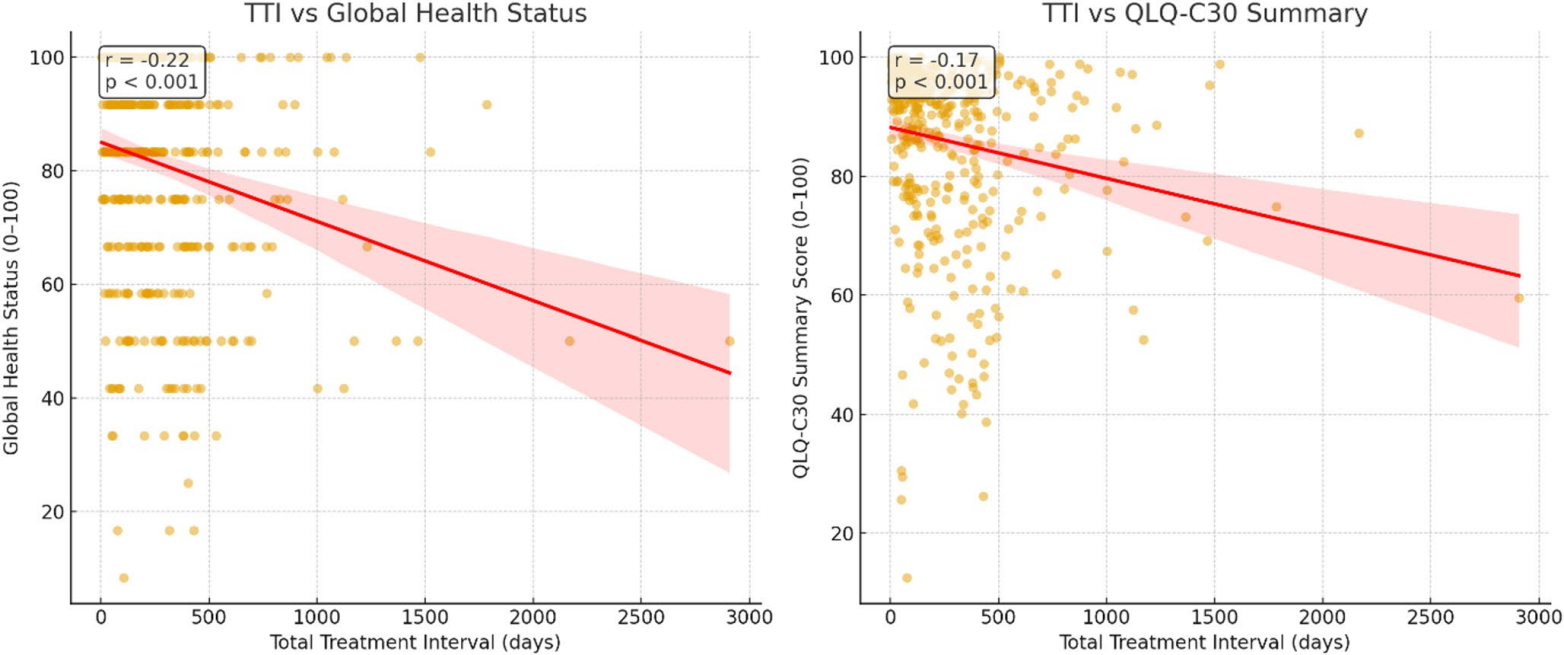
Correlation between total treatment interval and quality of life (EORTC QLQ-C30)

The total treatment interval (TTI) showed a significant inverse association with both measures of health-related quality of life. As TTI increased, scores on the GHS declined (Pearson *r* = − 0.22; *p* < 0.001), indicating poorer self-rated QoL. A similar pattern was observed for the QLQ-C30 summed score (Pearson *r* = − 0.17; *p* < 0.001). Longer total treatment intervals are associated with lower QoL (Fig. 2).

### Correlations between QoL scales and COST FACIT scales

**Fig. 3:**
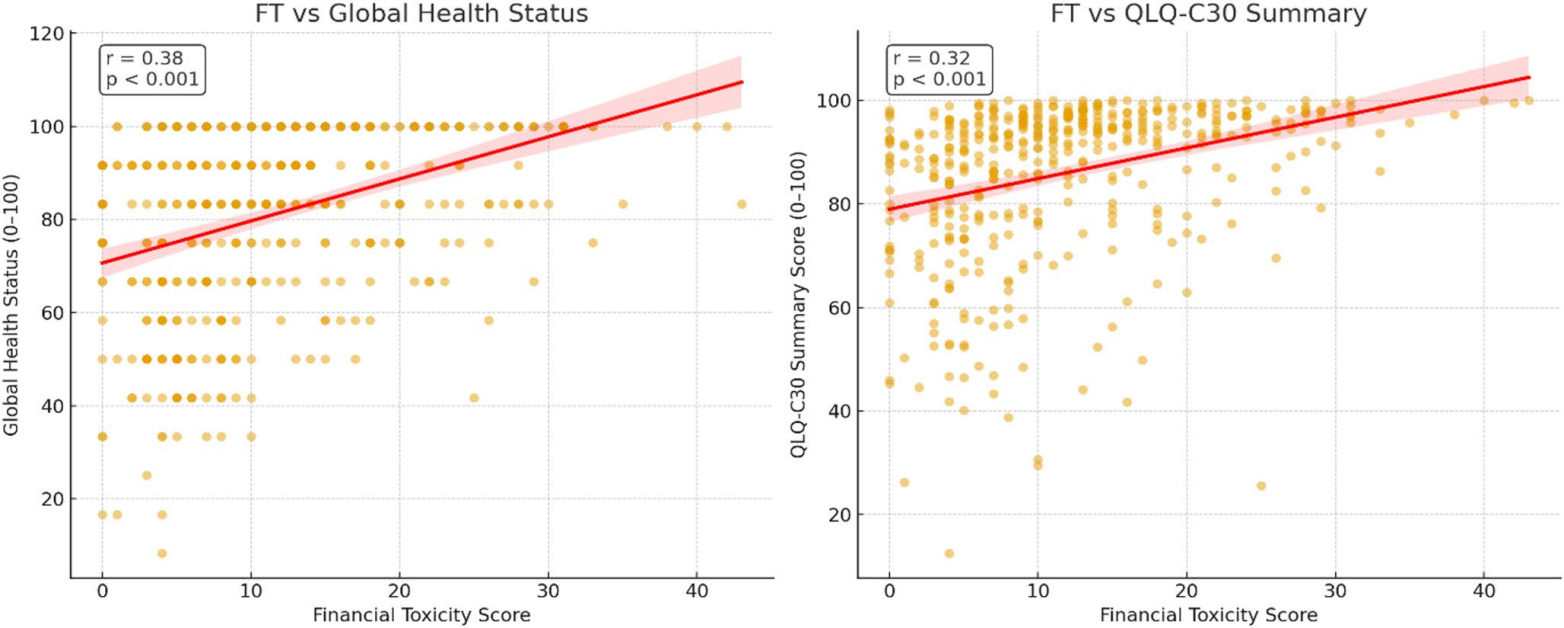
Correlation between financial toxicity (COST–FACIT scores) and quality of life domains (EORTC QLQ-C30)

Higher financial well-being, reflected by higher FT scores (lower financial hardship), was significantly associated with better QoL across both indicators (Fig. 3). FT scores showed a moderate positive correlation with GHS scores (*r* = 0.38, *p* < 0.001) and a slightly weaker significant association with the QLQ-C30 summed scores (*r* = 0.32, *p* < 0.001).

**Fig. 4:**
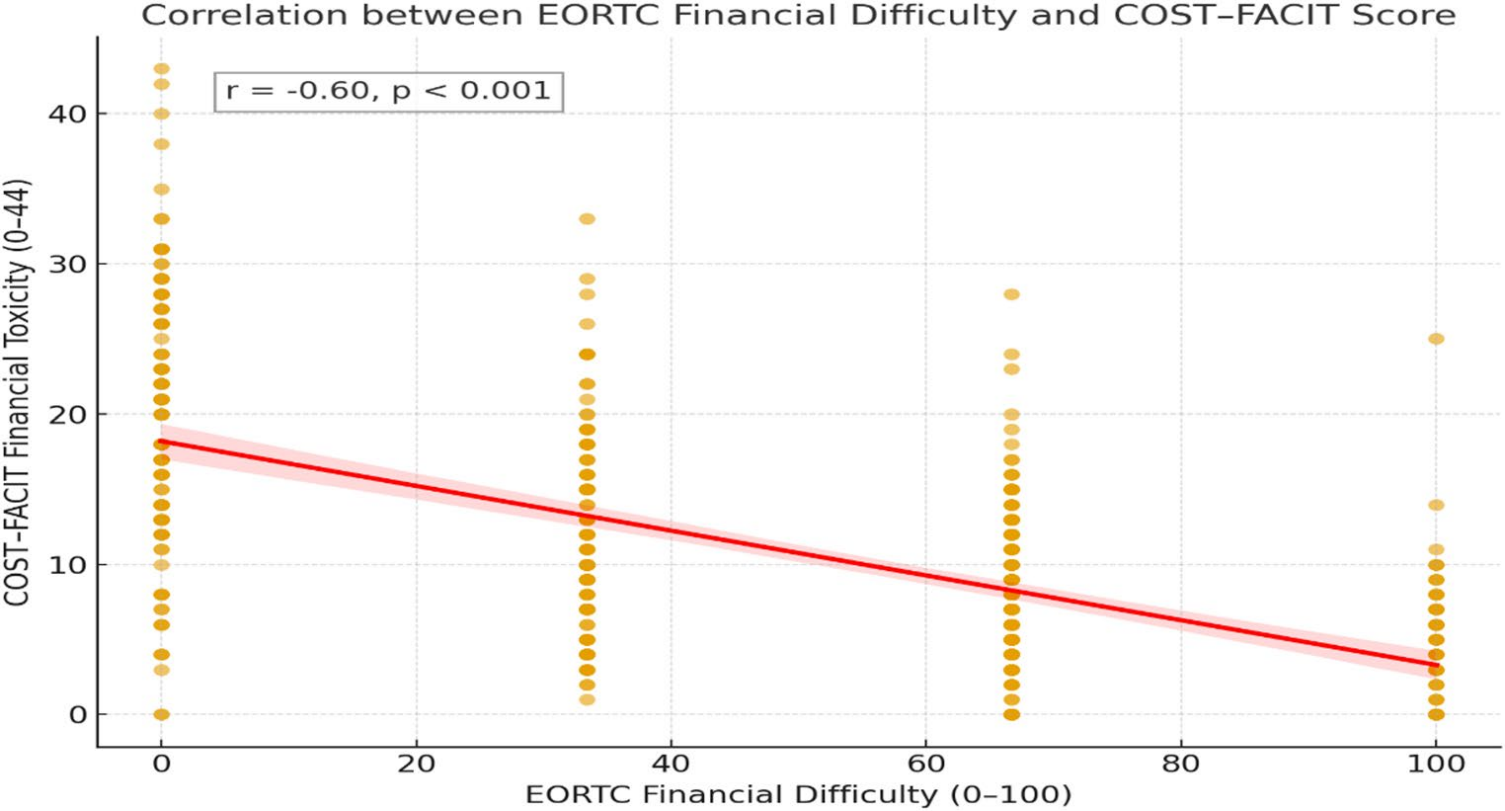
Correlation between the EORTC QLQ-C30 financial-difficulty item and COST–FACIT financial toxicity score among women with breast cancer in Ethiopia

The EORTC QLQ-C30 financial-difficulty item was strongly and inversely correlated with the COST-FACIT total score (Pearson’s *r* = − 0.60; *p* < 0.001), indicating that greater perceived financial strain corresponded to higher financial hardship. This finding supports strong evidence of convergent validity between the single-item EORTC measure and the multidimensional COST–FACIT financial toxicity scale (Fig. 4).

### Mediation model analysis

**Fig. 5:**
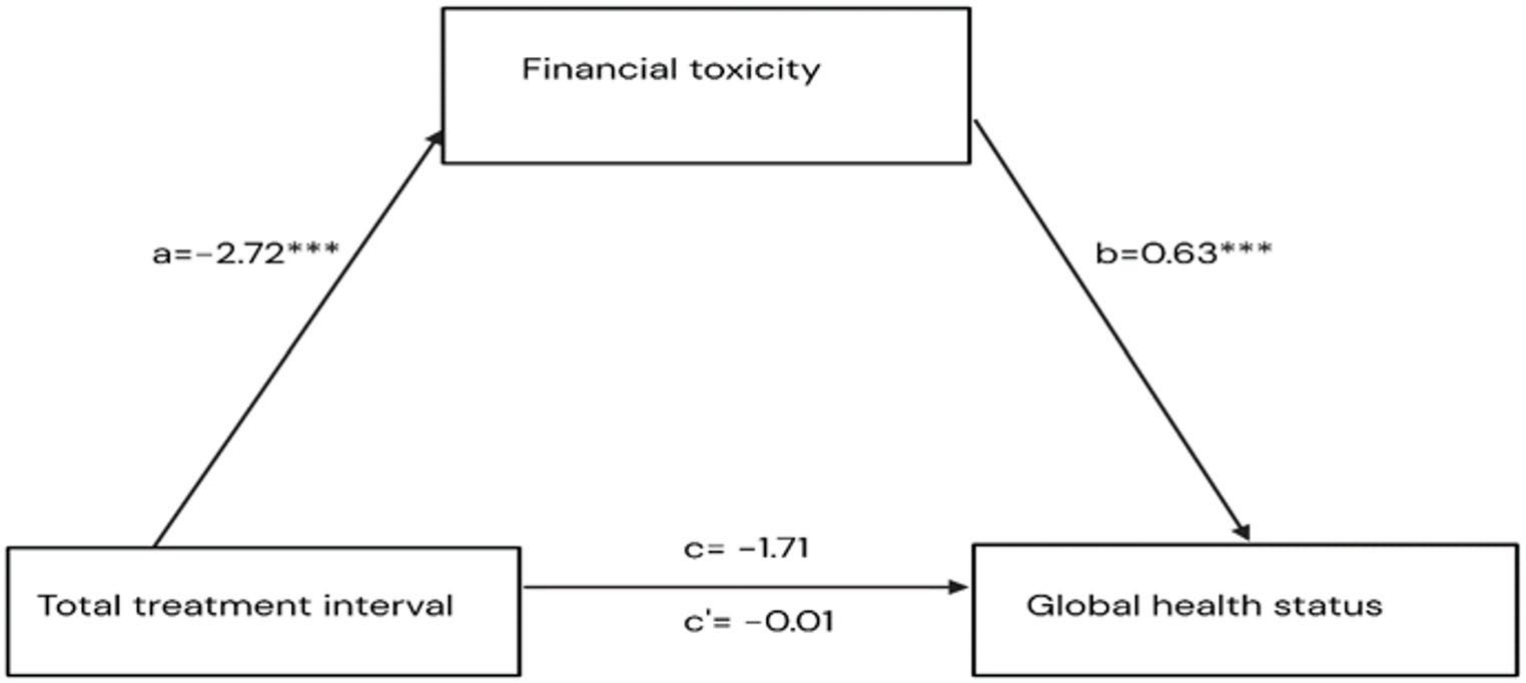
Model of the mediating effect of FT on the association between TTI and QOL (Global Health Status)

In the multivariable regression component of the mediation model, longer TTI (> 90 days) was significantly associated with lower FT scores, indicating higher financial hardship (path a: β = −2.72, *p* = 0.001). In turn, higher FT scores (indicating lower financial hardship) were positively associated with better GHS scores (path b: β = 0.63, *p* < 0.001) (Fig. 5). The indirect pathway from TTI to GHS scores via financial toxicity was significant (β = −1.70; 95% CI [− 2.94, − 0.66]). This indicates that the negative effect of delayed TTI on GHS operates indirectly through increased financial hardship. The direct pathway from TTI to QoL (path c′) was not significant (β = −0.01; *p* = 0.99), and the overall total effect of TTI on QoL was also non-significant (β = −1.71; 95% CI: −5.25 to 1.83; *p* = 0.34) (Table 2) (Fig. 5).

**Table 2:** The effects of TTI on global health status via financial toxicity

Path/Effect	Estimate (β)	SE	95% CI	*p*-value
Indirect effect (a×b)	−1.70	0.58	(− 2.94, − 0.66)	< 0.001
Direct effect (c′)	−0.01	1.76	(− 3.47, 3.45)	0.99
Total effect (c)	−1.71	1.80	(− 5.25, 1.83)	0.342

B unstandardized coefficient, CI confidence interval, SE standardized error

**Fig. 6:**
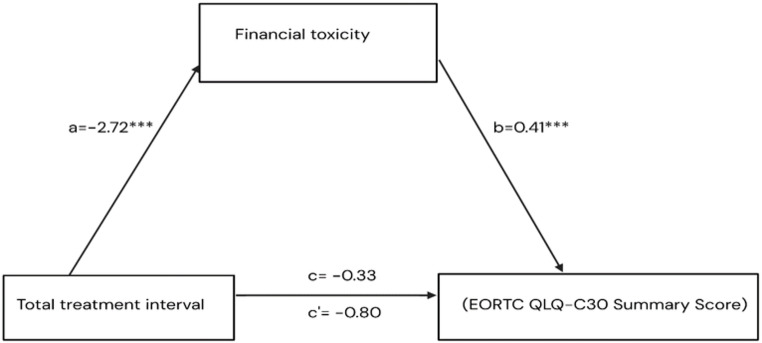
Model of the mediating effect of FT on the association between TTI and QOL (Summary Score)

When using the EORTC QLQ-C30 Summed Score as the outcome, the results were consistent, showing a significant indirect pathway linking TTI to the summed score through financial toxicity. longer TTI was significantly associated with higher financial hardship (β = −2.72, *p* = 0.001). Higher financial hardship (i.e., lower FT scores) was significantly associated with reduced overall QoL summed scores (β = 0.41, *p* < 0.001). The indirect effect of TTI on QoL summed scores via financial toxicity was statistically significant (β = −1.12, 95% CI − 2.03 to − 0.41). Whereas the direct (β = 0.80, *p* = 0.57) and total (β = −0.33, *p* = 0.82) effects were not (Table 3) (Fig. 6).

**Table 3:** The effects of TTI on quality of life mediated by financial toxicity (EORTC QLQ-C30 Summary Score)

Path/Effect	Estimate (β)	SE	95% CI	*p*-value
Indirect effect (a × b)	**−1.12**	**0.41**	**(− 2.03**, **− 0.41)**	**0.002**
Direct effect (c′)	0.80	1.40	(− 1.96, 3.55)	0.57
Total effect (c)	−0.33	1.42	(− 3.12, 2.46)	0.82

B unstandardized coefficient, CI confidence interval, SE standardized error

These results suggest that the detrimental influence of longer TTI on quality of life operates primarily through its impact on patients’ financial burden rather than through a direct pathway.

In the sensitivity analyses using continuous TTI as the exposure, no significant direct or total effects of TTI on either GHS or the EORTC QLQ-C30 Summed scores were observed, although both models consistently showed small but statistically significant indirect effects through financial toxicity (supplementary Table S2).

Moreover, the indirect effects of longer TTI on both functioning (β = −1.64, 95% CI − 2.87 to − 0.63) and symptom domain (β = −0.80, 95% CI − 1.53 to − 0.26) scores through financial toxicity were statistically significant, while the direct and total effects were not (Supplementary Tables S3).

### Moderated mediation model: cancer stage moderation

The indirect effect of TTI on GHS via financial toxicity was statistically significant in both early-stage (β = −1.62, 95% CI − 3.38 to − 0.15) and advanced-stage patients (β = −1.80, 95% CI − 3.55 to − 0.49). However, the index of moderated mediation was non-significant (β = −0.18, 95% CI − 2.40 to 1.96), indicating that the strength of the indirect effect did not differ meaningfully by cancer stage. That is, financial toxicity mediates the association between TTI and QoL similarly in both early and advanced disease (Table 4).

**Table 4:** Effect of TTI on global health status mediated by financial toxicity, moderated by cancer stage

Path/Effect	Estimate(β)	SE	95% CI	*p*-value
Conditional indirect effect (Stage I–II)	−1.62	0.82	(− 3.38, − 0.15)	< 0.001
Conditional indirect effect (Stage III–IV)	−1.80	0.78	(− 3.55, − 0.49)	< 0.001
Index of moderated mediation	−0.18	1.09	(− 2.40, 1.96)	0.86

Similarly, the indirect effects of TTI on QoL summed scores through financial toxicity were significant for both early-stage (β = −0.88, 95% CI − 2.05 to − 0.06) and advanced-stage patients (β = −1.38, 95% CI − 2.76 to − 0.36). However, the index of moderated mediation was non-significant (Index = − 0.50, 95% CI − 2.07 to 0.99), indicating that cancer stage did not significantly modify the strength of this indirect effect (Table 5).

**Table 5:** Moderated Mediation of TTI on QoL Summary Score via Financial Toxicity by Cancer Stage

Path/Effect	Estimate (β)	SE	95% CI (LL, UL)	*p*-value
Conditional indirect effect (Stage I–II)	−0.88	0.51	(− 2.05, − 0.06)	< 0.001
Conditional indirect effect (Stage III–IV)	−1.38	0.61	(− 2.76, − 0.36)	< 0.001
Index of moderated mediation	−0.50	0.77	(− 2.07, 0.99)	0.63

The sensitivity tests revealed similar patterns of results (Supplementary Tables S3). The indirect effects of TTI on QoL remained significant across both early and advanced-stage disease groups for functioning (Stage I–II: β = −1.35, 95% CI − 2.94 to − 0.12; Stage III–IV: β = −1.94, 95% CI − 3.81 to − 0.53) and symptom domains (Stage I–II: β = −0.58, 95% CI − 1.53 to 0.00; Stage III–IV: β = −1.03, 95% CI − 2.13 to − 0.24). However, the indices of moderated mediation were non-significant in both models (Functioning: β = −0.60, 95% CI − 2.74 to 1.44; Symptom: β = −0.45, 95% CI − 1.71 to 0.73. Cancer stage did not moderate the effects of TTI on either the functioning or symptom domains.

## Discussion

This multicentre study among 456 women with breast cancer in Ethiopia found that overall quality of life was relatively high, although emotional functioning and financial difficulties remained important concerns. Longer total treatment interval (TTI) was significantly associated with poorer quality of life, and financial toxicity was moderately correlated with QoL. Mediation analysis showed that financial toxicity mediated the relationship between prolonged TTI and reduced QoL, highlighting the economic consequences of delayed care. Socioeconomic disparities were evident, with rural residence, lower education, unemployment, and lower income associated with poorer QoL.

Women with breast cancer in this Ethiopian cohort reported unexpectedly high QoL scores, with a mean Global Health Status (GHS) of 81.2 and EORTC QLQ-C30 summed score of 85.8. These values exceed those typically reported in the literature. For example, a recent meta-analysis of 9,012 patients worldwide found an average EORTC QLQ-C30 QoL score of 64.7 [39]. Likewise, studies conducted in Ethiopia [28], Malaysia [40], and Morocco [41] reported mean GHS scores of 65, 69, and 68, respectively, among breast cancer patients. The relatively higher QoL in our sample may reflect the fact that over half were post-treatment survivors, who tend to regain functioning after completing therapy. Indeed, QoL improved significantly with treatment progress in our data: patients who had just begun treatment reported the lowest scores (mean GHS, 58), whereas those in the post-treatment phase had the highest QoL (mean GHS, 89), a pattern consistent with reports that survivors’ QoL tends to improve once acute treatment side effects subside [39, 42].

Most functional domains showed good preservation, with mean physical, role, cognitive, and social scores around 80 or higher. However, emotional functioning was notably lower, indicating that psychological well-being was more adversely affected than other QoL domains. Such a finding is common; for example, a South African study also found that the psychological domain was the lowest-scoring aspect of QoL among breast cancer patients [42]. Reduced emotional functioning may arise from the central caregiving role of women in Ethiopia, as illness disrupts their usual responsibilities. This disruption often creates heightened worry about their family and children’s future, contributing to poorer emotional well-being [43]. Fatigue and pain were the most frequently reported symptoms, although their severity was generally mild, with mean scores of 26 for fatigue and 20 for pain. Other symptoms were minimal; however, financial difficulties had a mean score of 44, indicating a substantial proportion of women experienced economic hardship related to their illness.

Our findings reveal pronounced socioeconomic and demographic disparities in quality of life. Urban patients had markedly better QoL than rural patients. This urban–rural gap likely reflects differences in access to care and support: rural patients often must travel long distances and face delays in obtaining treatment, which can worsen disease outcomes and QoL [44]. Besides, educational, occupational, and economic status also showed a strong gradient in QoL. Women with higher education, employment, and income reported better overall well-being than those with their counterparts. Consistent with this, a broad review of breast cancer in LMICs identified patient income as one of the most consistent predictors of QoL differences [45]. Other social factors (like marital status and age) can also influence QoL; for instance, we observed women aged 60 and above had significantly lower QoL than middle-aged women, and married patients had slightly better QoL than unmarried. These trends mirror findings from other settings that younger or middle-aged patients and those with spousal support often report higher QoL [42, 45].

This study also showed that a significant inverse correlation between TTI and QoL, as treatment intervals lengthened, QoL worsened. We also found a moderate positive correlation between FT scores and QoL. Which means lower financial hardship is strongly associated with better QoL. This magnitude of association is very much in line with observations from other settings in USA and Greek [46, 47].

Importantly, our analysis indicates that financial toxicity appears to play a key mediating role in the link between prolonged TTI and QoL. Mediation modeling confirmed that much of the effect of TTI on QoL is indirectly mediated through financial toxicity. In other words, when treatment is delayed, patients incur greater out-of-pocket costs (travel, lodging, lost income from extended illness, additional medical expenses), which in turn erode their quality of life. Delayed care in low-resource settings often forces patients to make repeated hospital trips, pay for interim treatments or supportive care, and potentially lose income due to prolonged illness, all contributing to financial toxicity [48]. Overall, our findings underscore that addressing the cost of care is central to maintaining patient quality of life and highlight financial toxicity as a key actionable mediator in the link between timely treatment and quality of life outcomes.

Interestingly, cancer stage at diagnosis did not significantly moderate the observed relationships between TTI, FT and QoL. This suggests that both early-stage and advanced-stage patients are vulnerable to the financial consequences of delay, and in both groups financial hardship similarly mediates QoL outcomes. In other words, the detrimental effect of longer TTI (mediated by FT) was evident regardless of stage of cancer. This may seem counterintuitive, but it has plausible explanations. First, in our study most women were already post-treatment and functionally recovered; at this stage, any residual QoL differences due solely to initial stage might be muted. Second, the range of stages in our sample was skewed (majority Stage II–III), which may have limited contrast.

In summary, these findings highlight that improving the quality of life of women with breast cancer in Ethiopia requires an integrated approach that extends beyond medical treatment. Clinicians should routinely assess psychosocial and financial well-being, provide emotional and social support, and connect economically vulnerable patients to available resources. At the policy level, strengthening financial risk protection, through expanded insurance coverage, treatment subsidies, and travel assistance, is critical to reduce financial toxicity and the urban–rural disparity in care. Efforts to shorten treatment delays by investing in cancer care infrastructure, early-detection programs, and standardized service delivery across hospitals could mitigate disease progression, prevent excessive out-of-pocket spending, and improve outcomes. By ensuring timely treatment and easing the financial burden of care, healthcare systems in Ethiopia and similar settings can significantly enhance the quality of life and well-being of women facing breast cancer. Future research should evaluate interventions that alleviate financial hardship, enhance emotional support, and track long-term QoL trajectories to inform equitable and sustainable breast-cancer care strategies in Ethiopia and other low-resource settings.

This study has several limitations. First, its cross-sectional design limits the ability to establish causality between treatment delay, financial toxicity, and quality of life (QoL). Second, reliance on patient self-report for some variables, including symptom detection and financial data, may introduce recall or reporting bias. Third, while the study included three major oncology centres, the sample may not fully represent breast cancer patients in remote or less-resourced regions of Ethiopia. Fourth, potential confounding factors such as social support, coping mechanisms, and psychological comorbidities were not assessed but may influence QoL outcomes. Lastly, the findings may not be generalizable to male breast cancer patients or those with recurrent disease, as the study focused on women with primary breast cancer in the curative phase.

## Conclusion

Overall QoL scores in our cohort were relatively high, but significant challenges persisted in financial difficulties. This study highlights that delays in treatment initiation adversely affect the quality of life of Ethiopian women with breast cancer, primarily through increased financial toxicity. Notably, this indirect effect held true regardless of cancer stage at diagnosis, underscoring that both early- and late-stage patients suffer the QoL consequences of delayed care through financial burden. These findings highlight the importance of timely treatment and financial risk protection to improve breast cancer outcomes in low-resource settings.

## Supplementary Information

Below is the link to the electronic supplementary material.

Supplementary Material 1

Supplementary Material 2

Supplementary Material 3

## Data Availability

The datasets generated and/or analysed during this study are not publicly available due to the inclusion of potentially identifiable health information. Data may be made available upon reasonable request, subject to approval by the La Trobe University Human Research Ethics Committee. Interested researchers should contact Professor George Liu ([C.Liu@latrobe.edu.au] (mailto: C.Liu@latrobe.edu.au)) or the Ethics Committee ([humanethics@latrobe.edu.au] (mailto: humanethics@latrobe.edu.au)).

## Abbreviations

NCCP: National Cancer Control Plan
OOP: Out-of-pocket
COST–FACIT: Comprehensive Score for Financial Toxicity – Functional Assessment of Chronic Illness Therapy

## References

[1] Zhang, Y., Ji, Y., Liu, S., Li, J., Wu, J., Jin, Q., Liu, X., Duan, H., Feng, Z., Liu, Y., & Zhang, Y. (2025). Global burden of female breast cancer: New estimates in 2022, temporal trend and future projections up to 2050 based on the latest release from GLOBOCAN. *Journal of the National Cancer Center,**5*(3), 287–296. 40693239 10.1016/j.jncc.2025.02.002 PMC12276554

[2] Tafese, A. M., Fentie, M. T., Seifu, B. L., Asnake, A. A., Dirbaba, B. D., Jara, A. G., Asare, E. T., & George, B. (2025). Breast cancer survival rates and determinants in Ethiopia: A systematic review and meta-analysis of longitudinal studies. *BMC Cancer,**25*(1), 1263. 40760653 10.1186/s12885-025-14705-9 PMC12323059

[3] Ssentongo, P., Lewcun, J. A., Candela, X., Ssentongo, A. E., Kwon, E. G., Ba, D. M., Oh, J. S., Amponsah-Manu, F., McDonald, A. C., Chinchilli, V. M., & Soybel, D. I. (2019). Regional, racial, gender, and tumor biology disparities in breast cancer survival rates in Africa: A systematic review and meta-analysis. *PLoS ONE,**14*(11), e0225039. 31751359 10.1371/journal.pone.0225039 PMC6872165

[4] Francies, F. Z., Hull, R., Khanyile, R., & Dlamini, Z. (2020). Breast cancer in low-middle income countries: Abnormality in splicing and lack of targeted treatment options. *American journal of cancer research,**10*(5), 1568–1591. 32509398 PMC7269781

[5] Pruthi, D. S., Ahmad, M., Gupta, M., Bansal, S., Nautiyal, V., & Saini, S. (2018). Assessment of quality of life in resectable gastric cancer patients undergoing chemoradiotherapy as adjuvant treatmentLetter to the Editor. *South Asian Journal of Cancer,**7*(01), 16–20. 29600226 10.4103/sajc.sajc_196_17 PMC5865087

[6] Mason, A., Juyal, R., Das, S. C., Shikha, D., Saini, S., & Semwal, J. (2019). Prevalence and correlates of psychological distress among cancer patients in a tertiary care hospital in northern India. *International Journal Of Community Medicine And Public Health,**6*(5), 2223–2228.

[7] Park, J., Rodriguez, J. L., O’Brien, K. M., Nichols, H. B., Hodgson, M. E., Weinberg, C. R., & Sandler, D. P. (2021). Health-related quality of life outcomes among breast cancer survivors. *Cancer,**127*(7), 1114–1125. 33237602 10.1002/cncr.33348 PMC8035208

[8] Breek, K., Abuhalima, D., Salameh, H., Al-Jabi, S. W., & Zyoud, S. E. H. (2025). Self-reported side effects of breast cancer treatment and its impact on quality of life: A multicenter cross-sectional study in a low-and middle-income country. *BMC Cancer,**25*(1), 975. 40452019 10.1186/s12885-025-14381-9 PMC12128375

[9] Montazeri, A., Milroy, R., Hole, D., McEwen, J., & Gillis, C. R. (2003). How quality of life data contribute to our understanding of cancer patients’ experiences? A study of patients with lung cancer. *Quality of Life Research,**12*(2), 157–166. 12639062 10.1023/a:1022232624891

[10] Montazeri, A. (2008). Health-related quality of life in breast cancer patients: A bibliographic review of the literature from 1974 to 2007. *Journal of Experimental & Clinical Cancer Research,**27*(1), 32. 18759983 10.1186/1756-9966-27-32 PMC2543010

[11] Franzoi, M. A., Di Meglio, A., Michiels, S., Gillanders, E., Gaudin, C., Martin, A. L., & Vaz-Luis, I. (2024). Patient-reported quality of life 6 years after breast cancer. *JAMA Network Open,**7*(2), e240688. 38421653 10.1001/jamanetworkopen.2024.0688 PMC10905303

[12] Paleri, V., Wight, R. G., Silver, C. E., Haigentz, M., Jr., Takes, R. P., Bradley, P. J., Rinaldo, A., Sanabria, A., Bień, S., & Ferlito, A. (2010). Comorbidity in head and neck cancer: A critical appraisal and recommendations for practice. *Oral Oncology,**46*(10), 712–719. 20850371 10.1016/j.oraloncology.2010.07.008

[13] Sabir, K., Sajid, J., Butt, N. I., Waris, B., Zahra, A., Shoaib, M., & Butt, N. I. (2025). Clinical profile and outcome of patients with advanced hepatocellular carcinoma (HCC) taking sorafenib or lenvatinib: Real-world experience from a low-middle resource country. *Cureus*. 10.7759/cureus.83681 40486326 10.7759/cureus.83681 PMC12143940

[14] Zhao, Q., Kuang, Y., Yuan, X., Sun, Y., Zhu, Z., Zhu, J., Gu, H., & Xing, W. (2025). Financial toxicity and quality of life among older adults with cancer: A moderated mediation model of loneliness and social support. *Supportive Care in Cancer,**33*(8), 680. 40640458 10.1007/s00520-025-09718-w

[15] McKenzie, F., Zietsman, A., Galukande, M., Anele, A., Adisa, C., Cubasch, H., Parham, G., Anderson, B. O., Abedi-Ardekani, B., Schuz, J., & dos Santos Silva, I. (2016). African Breast Cancer—Disparities in Outcomes (ABC-DO): Protocol of a multicountry mobile health prospective study of breast cancer survival in sub-Saharan Africa. *British Medical Journal Open,**6*(8), e011390. 10.1136/bmjopen-2016-011390 PMC5013398 27554102

[16] Williams, F. (2015). Assessment of breast cancer treatment delay impact on prognosis and survival: A look at the evidence from systematic analysis of the literature. *Journal of Cancer Biology & Research,**3*(4), 1071. 34258389 PMC8274552

[17] Konieczny, M., Cipora, E., Roczniak, W., Babuśka-Roczniak, M., & Wojtaszek, M. (2020). Impact of time to initiation of treatment on the quality of life of women with breast cancer. *International Journal of Environmental Research and Public Health,**17*(22), 8325. 10.3390/ijerph17228325 33187071 10.3390/ijerph17228325 PMC7696805

[18] WHO. (2023). *Global breast cancer initiative implementation framework: assessing, strengthening and scaling up of services for the early detection and management of breast cancer: Executive summary*. Available from: https://www.who.int/publications/i/item/9789240067134?utm_source=chatgpt.com

[19] Kibret, A. A., Jiang, H., Yang, H., & Liu, C. (2024). Patient journey and timeliness of care for patients with breast cancer in Africa: A scoping review. *British Medical Journal Open,**15*(8), e098087. 10.1136/bmjopen-2024-098087 PMC12406880 40887113

[20] Hoveling, L. A., Schuurman, M., Siesling, S., van Asselt, K. M., & Bode, C. (2025). Diagnostic delay in women with cancer: What do we know and which factors contribute? *The Breast,**80*, 104427. 10.1016/j.breast.2025.104427 39987718 10.1016/j.breast.2025.104427 PMC11904510

[21] Ngan, T. T., Van Minh, H., Donnelly, M., & O’Neill, C. (2021). Financial toxicity due to breast cancer treatment in low-and middle-income countries: Evidence from Vietnam. *Supportive Care in Cancer,**29*(11), 6325–6333. 33860362 10.1007/s00520-021-06210-z PMC8464564

[22] Bhangdia, K., Natarajan, A., Rudolfson, N., Verguet, S., Castro, M. C., Dusengimana, J. M., Shyirambere, C., Schleimer, L. E., Shulman, L. N., Umwizerwa, A., & Kigonya, C. (2025). The association of travel distance and other patient characteristics with breast cancer stage at diagnosis and treatment completion at a rural Rwandan cancer facility. *BMC Cancer,**25*(1), 146. 39865262 10.1186/s12885-025-13489-2 PMC11771020

[23] Jalali, F. S., Seif, M., Jafari, A., Zangouri, V., Keshavarz, K., & Ravangard, R. (2023). Factors affecting the economic burden of breast cancer in southern Iran. *BMC Health Services Research,**23*(1), 1332. 38041035 10.1186/s12913-023-10346-5 PMC10691120

[24] Noronha, V., Tongaonkar, A., Pillai, A., Rao, A. R., Kumar, A., Sehgal, A., Basu, R., Ramaswamy, A., Dhekale, R., Daptardar, A., & Sonkusare, L. (2025). Prevalence and impact of financial toxicity in older patients with cancer: A prospective observational study in India. *Supportive Care in Cancer,**33*(5), 416. 40278900 10.1007/s00520-025-09252-9 PMC12031899

[25] Sakti, V. V., Danaee, M., Yip, C. H., Bustamam, R. S., Saad, M., Gan, G. G., Tan, J., Lim, Y. N., Chong, F. L., Munisamy, M., & Farid, F. M. (2023). Financial toxicity following cancer in a middle-income country with a pluralistic health system: Validation of the COST questionnaire. *Cancer Care Research Online,**3*(3), e044.

[26] Tran, G., & Zafar, S. Y. (2018). Financial toxicity and implications for cancer care in the era of molecular and immune therapies. *Annals of Translational Medicine,**6*(9), 166. 29911114 10.21037/atm.2018.03.28 PMC5985271

[27] Aaronson, N. K. (1993). The European Organization for Research and Treatment of Cancer QLQ-C30: A quality-of-life instrument for use in international clinical trials in oncology. *JNCI Journal of the National Cancer Institute,**85*(5), 365–376. 8433390 10.1093/jnci/85.5.365

[28] Getu, M. A., Chen, C., Wang, P., Kantelhardt, E. J., & Addissie, A. (2022). Quality of life and its influencing factors among breast cancer patients at Tikur Anbessa specialised hospital, Addis Ababa, Ethiopia. *BMC Cancer,**22*(1), 897. 35978281 10.1186/s12885-022-09921-6 PMC9382842

[29] Alem, T., Nigatu, D., Birara, A., Fetene, T., & Giza, M. (2024). Quality of life of breast cancer patients in Amhara region, Ethiopia: A cross-sectional study. *PLoS ONE,**19*(6), e0305263. 38935776 10.1371/journal.pone.0305263 PMC11210875

[30] Belhaj Haddou, M., Igarramen, T., Khouchani, M., & Elkhoudri, N. (2024). Determinants of health-related quality of life in breast cancer patients: A comprehensive study in Marrakech, Morocco. *The Open Public Health Journal*, *17*(1).

[31] Cserni, G., Chmielik, E., Cserni, B., & Tot, T. (2018). The new TNM-based staging of breast cancer. *Virchows Archiv,**472*(5), 697–703. 29380126 10.1007/s00428-018-2301-9

[32] Van Oostrom, S. H., Picavet, H. S., Van Gelder, B. M., Lemmens, L. C., Hoeymans, N., Van Dijk, C. E., Verheij, R. A., Schellevis, F. G., & Baan, C. A. (2012). Multimorbidity and comorbidity in the Dutch population – Data from general practices. *BMC Public Health,**12*(1), 715. 22935268 10.1186/1471-2458-12-715 PMC3490727

[33] WHO, W.H.O.N.d. (2023). *Noncommunicable diseases*. Available from: https://www.who.int/health-topics/noncommunicable-diseases#tab=tab_1

[34] Ayana, B. A., Negash, S., Yusuf, L., Tigeneh, W., & Haile, D. (2016). Reliability and validity of amharic version of EORTC QLQ-C 30 questionnaire among gynecological cancer patients in Ethiopia. *PLoS ONE,**11*(6), e0157359. 27304066 10.1371/journal.pone.0157359 PMC4909272

[35] Fayers, P., et al. (2001). *EORTC QLQ-C30 scoring manual*. European Organisation for research and treatment of cancer.

[36] Fayers, P. M., & Machin, D. (2015). *Quality of life: The assessment, analysis and reporting of patient-reported outcomes*. John Wiley & Sons.

[37] Giesinger, J. M., Kieffer, J. M., Fayers, P. M., Groenvold, M., Petersen, M. A., Scott, N. W., Sprangers, M. A., Velikova, G., Aaronson, N. K., EORTC Quality of Life Group. (2016). Replication and validation of higher order models demonstrated that a summary score for the EORTC QLQ-C30 is robust. *Journal of Clinical Epidemiology,**69*, 79–88. 26327487 10.1016/j.jclinepi.2015.08.007

[38] Hayes, A. F. (2017). *Introduction to mediation, moderation, and conditional process analysis: A regression-based approach*. Guilford publications.

[39] Biparva, A. J., Raoofi, S., Rafiei, S., Kan, F. P., Kazerooni, M., Bagheribayati, F., Masoumi, M., Doustmehraban, M., Sanaei, M., Zarabi, F., & Raoofi, N. (2024). Global quality of life in breast cancer: systematic review and meta-analysis. *BMJ Supportive & Palliative Care,**13*(e3), e528–e536. 10.1136/bmjspcare-2022-003642 PMC10850719 35710706

[40] Shin, K. N. L., Mun, C. Y., & Shariff, Z. M. (2020). Nutrition indicators, physical function, and health-related quality of life in breast cancer patients. *Asian Pacific Journal of Cancer Prevention: APJCP,**21*(7), 1939. 32711419 10.31557/APJCP.2020.21.7.1939 PMC7573431

[41] El Fakir, S., El Rhazi, K., Zidouh, A., Bennani, M., Benider, A., Errihani, H., Mellass, N., Bekkali, R., & Nejjari, C. (2016). Health-related quality of life among breast cancer patients and influencing factors in Morocco. *Asian Pacific Journal of Cancer Prevention: APJCP,**17*(12), 5063. 28122435 10.22034/APJCP.2016.17.12.5063 PMC5454637

[42] Wilkinson, R., & Smith, L. (2024). Quality of life in female breast cancer patients and survivors in a South African Municipality. *Breast Cancer,**18*, 11782234241282520. 39391809 10.1177/11782234241282519 PMC11465291

[43] Hassen, A. M., Taye, G., Gizaw, M., & Hussien, F. M. (2019). Quality of life and associated factors among patients with breast cancer under chemotherapy at Tikur Anbessa specialized hospital, Addis Ababa, Ethiopia. *PLoS ONE,**14*, e0222629. 31539399 10.1371/journal.pone.0222629 PMC6754151

[44] Parikh-Patel, A., Morris, C. R., Kizer, K. W., Wun, T., & Keegan, T. H. (2021). Urban-rural variations in quality of care among patients with cancer in California. *American Journal of Preventive Medicine,**61*, e279–e288. 34404553 10.1016/j.amepre.2021.05.021

[45] Ngo, N. T., Nguyen, H. T., Nguyen, P. T., Vo, T. T., Phung, T. L., Pham, A. G., Vo, T. V., Dang, M. T., Nguyen Le Bao, T., & Duong, K. N. (2023). Health-related quality of life in breast cancer patients in low-and-middle-income countries in Asia: A systematic review. *Frontiers in Global Women’s Health,**4*, 1180383. 37389285 10.3389/fgwh.2023.1180383 PMC10304018

[46] Pitis, A., Diamantopoulou, M., Panagiotou, A., Papageorgiou, D., & Tzavella, F. (2025). Financial toxicity and its association with the quality of life of Greek patients with cancer: A cross-sectional study. *Nursing Reports,**15*(2), 67. 39997803 10.3390/nursrep15020067 PMC11858100

[47] De Souza, J. A., Yap, B. J., Wroblewski, K., Blinder, V., Araújo, F. S., Hlubocky, F. J., Nicholas, L. H., O’Connor, J. M., Brockstein, B., Ratain, M. J., & Daugherty, C. K. (2017). Measuring financial toxicity as a clinically relevant patient-reported outcome: The validation of the COmprehensive Score for financial Toxicity (COST). *Cancer,**123*, 476–484. 27716900 10.1002/cncr.30369 PMC5298039

[48] Mulugeta, C., Emagneneh, T., Kumie, G., Ejigu, B., & Alamrew, A. (2024). Delayed presentation of breast cancer patients and contributing factors in East Africa: Systematic review and meta-analysis. *PLoS ONE,**19*, e0309792. 39527621 10.1371/journal.pone.0309792 PMC11554124

