# Supplementary material for: Total treatment interval and quality of life of women living with breast cancer in Ethiopia: the mediating role of financial toxicity
Source: Qual Life Res. 2026 Apr 1;35(5):113. doi: 10.1007/s11136-026-04225-9 (PMC13043552; doi:10.1007/s11136-026-04225-9)
Supplement: Supplementary file 1 — Supplementary Material 1 [file 11136_2026_4225_MOESM1_ESM.docx]

Supplementary table S3

The indirect effect through financial toxicity was significant for both early- and advanced-stage disease, but the index of moderated mediation was non-significant, indicating that the strength of this mediation pathway did not differ by stage.

Table1: PROCESS Model 59 results testing the moderating effect of cancer stage on the indirect association between TTI and global quality of life.

| **Path / Effect** | **Estimate (β)** | **SE** | **95 % CI (LL, UL)** | **p-value** |
| --- | --- | --- | --- | --- |
| **Indirect effect (overall)** | −1.70 | 0.58 | (−2.94, −0.66) | <0.001 |
| **Conditional indirect (Stage I–II)** | −1.62 | 0.82 | (−3.38, −0.15) | <0.001 |
| **Conditional indirect (Stage III–IV)** | −1.80 | 0.78 | (−3.55, −0.49) | <0.001 |
| **Direct effect (overall)** | −0.01 | 1.76 | (−3.47, 3.45) | 0.99 |
| **Conditional direct (Stage I–II)** | −0.89 | 2.44 | (−5.69, 3.91) | 0.71 |
| **Conditional direct (Stage III–IV)** | 0.97 | 2.50 | (−3.94, 5.89) | 0.70 |
| **Index of moderated mediation** | −0.18 | 1.09 | (−2.40, 1.96) | 0.86 |

The results showed that the indirect effects of treatment delay on quality of life through financial toxicity were significant for both early-stage (β = −0.88, 95% CI −2.05 to −0.06) and advanced-stage patients (β = −1.38, 95% CI −2.76 to −0.36). However, the index of moderated mediation was non-significant (Index = −0.50, 95% CI −2.07 to 0.99), indicating that cancer stage did not significantly modify the strength of this indirect effect.

Table 2: Moderated mediation of the association between treatment delay and overall quality of life (QLQ-C30 summary) through financial toxicity, by cancer stage.

| **Path / Effect** | **Estimate (β)** | **SE** | **95% CI (LL, UL)** | **p-value** |
| --- | --- | --- | --- | --- |
| **Indirect effect (overall)** | −1.12 | 0.41 | (−2.03, −0.41) | 0.002 |
| **Conditional indirect (Stage I–II)** | −0.88 | 0.51 | (−2.05, −0.06) | <0.001 |
| **Conditional indirect (Stage III–IV)** | −1.38 | 0.61 | (−2.76, −0.36) | <0.001 |
| **Direct effect (overall)** | 0.80 | 1.40 | (−1.96, 3.55) | 0.57 |
| **Conditional direct (Stage I–II)** | 0.43 | 1.95 | (−3.39, 4.26) | 0.82 |
| **Conditional direct (Stage III–IV)** | 1.10 | 1.99 | (−2.81, 5.01) | 0.58 |
| **Index of moderated mediation** | −0.50 | 0.77 | (−2.07, 0.99) | 0.63 |
