# Supplementary material for: Total treatment interval and quality of life of women living with breast cancer in Ethiopia: the mediating role of financial toxicity
Source: Qual Life Res. 2026 Apr 1;35(5):113. doi: 10.1007/s11136-026-04225-9 (PMC13043552; doi:10.1007/s11136-026-04225-9)
Supplement: Supplementary file 2 — Supplementary Material 2 [file 11136_2026_4225_MOESM2_ESM.docx]

**Supplementary file 2**

When total treatment interval was modelled as a continuous variable, mediation analysis showed a significant indirect effect through financial toxicity (β = −0.0013, 95% CI −0.0028, −0.0001, p = 0.030). The direct effect of TTI on quality of life was not significant (β = −0.0031, 95% CI −0.0082, 0.0019, p = 0.22), and the total effect was not also significant (−0.0044, 95% CI –0.0097 to 0.0008, p = 0.097) (Supplementary Table 2).

**Table 1: Direct, Indirect, and Total Effects of TTI (continuous TTI) on QoL Outcomes**

| **Outcomes** | **Path / Effect** | **β (Estimate)** | **SE** | **95 % CI (LL, UL)** | ***p*-value** |
| --- | --- | --- | --- | --- | --- |
| **Global Health (GH)** | Indirect effect (a×b) | **−0.0013** | 0.0007 | ( −0.0028, −0.0001) | **0.03** |
|  | Direct effect (c′) | −0.0031 | 0.0026 | ( −0.0082, 0.0019) | 0.22 |
|  | Total effect (c) | −0.0044 | 0.0027 | ( −0.0097, 0.0008) | 0.10 |
| **QLQ-C30 Summary Score** | Indirect effect (a×b) | **−0.0008** | 0.0005 | ( −0.0019, −0.0001) | **0.04** |
|  | Direct effect (c′) | −0.0012 | 0.0021 | ( −0.0052, 0.0029) | 0.56 |
|  | Total effect (c) | −0.0020 | 0.0021 | ( −0.0062, 0.0021) | 0.33 |
