# Supplementary material for: Total treatment interval and quality of life of women living with breast cancer in Ethiopia: the mediating role of financial toxicity
Source: Qual Life Res. 2026 Apr 1;35(5):113. doi: 10.1007/s11136-026-04225-9 (PMC13043552; doi:10.1007/s11136-026-04225-9)
Supplement: Supplementary file 3 — Supplementary Material 3 [file 11136_2026_4225_MOESM3_ESM.docx]

**Supplementary Table S1**

**Table 1: Comparison of means of functional scales according to patient characteristics.**

| Characteristic | PF | RF | EF | CF | SF |
| --- | --- | --- | --- | --- | --- |
| Mean (SD) | 82.0 (17.9) | 81.2 (24.2) | 77.0 (19.7) | 88.3 (21.0) | 80.2 (23.9) |
| Age (Years) |  |  |  |  |  |
| <40 | 82.8 (16.7) | 79.9 (23.3) | 73.3 (19.7) | 89.5 (19.3) | 77.3 (24.0) |
| 40–59 | 83.5 (17.7) | 83.7 (23.0) | 79.6 (18.1) | 89.4 (19.3) | 82.4 (23.3) |
| ≥60 | 72.8 (19.7) | 73.5 (29.8) | 77.2 (24.1) | 79.2 (29.9) | 78.9 (25.5) |
| p | <0.001 | 0.024 | 0.002 | 0.076 | 0.045 |
| Residence |  |  |  |  |  |
| Urban | 84.2 (16.1) | 84.6 (21.4) | 78.0 (18.8) | 91.0 (18.4) | 83.4 (20.2) |
| Rural | 75.6 (21.0) | 71.3 (28.8) | 73.9 (21.7) | 80.3 (25.5) | 70.9 (30.4) |
| p | <0.001 | <0.001 | 0.088 | <0.001 | <0.001 |
| Study site |  |  |  |  |  |
| Black Lion | 83.7 (15.9) | 84.9 (20.4) | 77.4 (17.8) | 94.4 (14.8) | 83.2 (19.6) |
| Hiwot Fana | 66.7 (25.3) | 63.3 (33.6) | 64.9 (24.2) | 69.2 (25.0) | 54.1 (30.1) |
| Jimma | 86.0 (12.9) | 82.9 (21.9) | 82.1 (18.1) | 86.3 (22.9) | 86.9 (19.7) |
| p | <0.001 | <0.001 | <0.001 | <0.001 | <0.001 |
| Marital status |  |  |  |  |  |
| Married | 83.3 (16.9) | 82.9 (24.0) | 77.8 (19.2) | 89.8 (19.9) | 81.6 (24.1) |
| Unmarried | 79.9 (19.3) | 78.4 (24.3) | 75.8 (20.4) | 85.9 (22.7) | 77.9 (23.5) |
| p | 0.106 | 0.017 | 0.321 | 0.074 | 0.023 |
| Occupational status |  |  |  |  |  |
| Employed | 85.3 (15.4) | 84.8 (21.4) | 79.4 (17.2) | 92.1 (16.7) | 84.2 (19.9) |
| Unemployed | 78.9 (19.5) | 77.8 (26.2) | 74.8 (21.5) | 84.7 (24.0) | 76.4 (26.7) |
| p | <0.001 | 0.002 | 0.050 | 0.0003 | 0.004 |
| Educational attainment |  |  |  |  |  |
| No formal education | 74.8 (20.7) | 72.9 (28.2) | 73.8 (21.8) | 82.7 (24.6) | 73.6 (28.0) |
| Primary & secondary | 85.2 (15.6) | 83.9 (21.6) | 77.6 (18.9) | 90.5 (19.3) | 82.3 (22.2) |
| Higher education | 86.6 (14.2) | 88.3 (18.9) | 81.0 (17.0) | 92.5 (16.6) | 86.1 (17.5) |
| p | <0.001 | <0.001 | 0.039 | 0.0002 | 0.0015 |
| Monthly household income (ETB) tertiles |  |  |  |  |  |
| ≤2,100 (T1) | 77.8 (19.1) | 75.7 (26.4) | 73.7 (20.0) | 86.2 (21.0) | 74.1 (24.8) |
| 2,101–3,254 (T2) | 83.3 (17.5) | 83.1 (23.2) | 75.6 (21.1) | 87.4 (22.6) | 81.4 (24.1) |
| 3,255–15,000 (T3) | 85.9 (15.7) | 86.0 (21.1) | 82.1 (17.0) | 91.6 (19.4) | 86.2 (21.1) |
| p | <0.001 | 0.0003 | 0.0002 | 0.0083 | <0.001 |
| Distance to the health facility (km) |  |  |  |  |  |
| <5 | 81.8 (17.8) | 81.3 (24.1) | 76.6 (19.9) | 88.3 (20.5) | 79.7 (24.1) |
| ≥5 | 82.9 (18.4) | 80.8 (24.8) | 78.6 (18.8) | 88.2 (22.8) | 81.9 (23.5) |
| p | 0.332 | 0.992 | 0.327 | 0.658 | 0.377 |
| Health insurance |  |  |  |  |  |
| Yes | 82.1 (18.3) | 80.9 (24.6) | 76.9 (19.9) | 87.9 (21.8) | 81.1 (24.2) |
| No | 81.7 (16.3) | 82.4 (22.8) | 77.6 (18.6) | 89.7 (17.7) | 76.7 (22.8) |
| p | 0.547 | 0.676 | 0.986 | 0.840 | 0.032 |
| Total Treatment Interval (TTI) |  |  |  |  |  |
| ≤90 days | 87.2 (16.8) | 88.8 (20.7) | 82.4 (19.0) | 90.2 (20.2) | 86.0 (20.8) |
| >90 days | 79.9 (17.9) | 78.0 (24.9) | 74.8 (19.5) | 87.5 (21.4) | 77.8 (24.7) |
| p | <0.001 | <0.001 | <0.001 | 0.180 | <0.001 |
| Family size |  |  |  |  |  |
| <2 | 83.5 (16.4) | 84.0 (21.3) | 78.6 (19.1) | 89.1 (22.0) | 81.9 (19.3) |
| 2–5 | 82.5 (17.3) | 82.3 (22.8) | 76.5 (19.6) | 89.1 (20.4) | 81.2 (23.1) |
| >6 | 77.6 (22.0) | 71.8 (31.7) | 77.3 (20.9) | 83.6 (22.1) | 72.8 (31.8) |
| p | 0.349 | 0.050 | 0.523 | 0.042 | 0.384 |
| Stage at diagnosis |  |  |  |  |  |
| Stage I | 84.3 (14.9) | 88.2 (14.1) | 82.8 (15.2) | 95.1 (11.4) | 86.3 (15.9) |
| Stage II | 85.2 (16.8) | 85.8 (21.1) | 78.8 (18.7) | 91.2 (19.3) | 84.6 (21.0) |
| Stage III | 82.7 (17.3) | 82.4 (24.2) | 77.9 (19.3) | 88.5 (19.9) | 80.6 (24.0) |
| Stage IV | 69.1 (18.9) | 60.8 (25.5) | 66.8 (22.2) | 76.9 (27.8) | 63.5 (27.0) |
| p | <0.001 | <0.001 | 0.0005 | <0.001 | <0.001 |
| Days since treatment initiation |  |  |  |  |  |
| Just started | 61.9 (23.0) | 48.8 (27.3) | 61.3 (29.7) | 67.9 (37.2) | 60.7 (35.0) |
| Early/Mid-treatment | 74.5 (19.6) | 67.8 (28.0) | 70.1 (21.2) | 83.3 (24.4) | 67.3 (27.8) |
| Late treatment | 78.8 (20.4) | 76.7 (25.0) | 73.5 (20.3) | 87.2 (22.7) | 77.7 (24.5) |
| Post-treatment | 87.4 (13.3) | 90.1 (16.4) | 82.0 (16.3) | 91.9 (16.3) | 87.5 (17.5) |
| p | <0.001 | <0.001 | <0.001 | 0.001 | <0.001 |
| Medically confirmed chronic illness |  |  |  |  |  |
| No | 82.7 (17.8) | 81.2 (24.1) | 76.4 (19.6) | 88.7 (20.9) | 80.5 (23.4) |
| Yes | 79.0 (18.3) | 81.0 (25.1) | 79.7 (20.0) | 86.8 (21.9) | 78.7 (26.0) |
| p | 0.035 | 1.000 | 0.081 | 0.474 | 0.711 |
| Treatment modality |  |  |  |  |  |
| Single modality | 69.2 (20.4) | 61.3 (28.0) | 68.7 (23.2) | 79.1 (28.1) | 65.1 (28.0) |
| Two modalities | 83.3 (17.1) | 83.4 (22.3) | 77.3 (18.6) | 90.1 (19.0) | 81.1 (22.7) |
| All three modalities | 89.2 (11.4) | 91.4 (16.1) | 84.1 (16.9) | 90.3 (18.6) | 90.9 (16.7) |
| p | <0.001 | <0.001 | <0.001 | 0.004 | <0.001 |
| Hospitalizations (past year) |  |  |  |  |  |
| 0–3 | 82.8 (17.2) | 81.6 (24.0) | 77.9 (18.9) | 89.2 (20.2) | 80.8 (23.7) |
| 4–7 | 60.8 (23.8) | 69.8 (28.0) | 54.2 (25.5) | 62.5 (28.2) | 64.6 (25.0) |
| p | <0.001 | 0.074 | <0.001 | <0.001 | 0.007 |
| BMI† (kg/m²) |  |  |  |  |  |
| Underweight (<18.5) | 69.6 (23.7) | 60.0 (33.3) | 68.3 (24.7) | 72.0 (29.5) | 65.3 (30.0) |
| Normal (18.5–24.9) | 82.8 (17.1) | 82.4 (22.6) | 77.0 (18.0) | 89.3 (20.0) | 80.5 (23.2) |
| Overweight (25–29.9) | 79.4 (19.4) | 79.2 (26.8) | 75.9 (24.4) | 88.4 (22.5) | 78.9 (25.2) |
| Obese (30–39.9) | 90.3 (9.6) | 90.3 (15.5) | 88.5 (16.6) | 90.3 (13.8) | 93.1 (14.7) |
| p | 0.001 | 0.001 | 0.001 | 0.007 | 0.001 |

*Note: Abbreviations: PF, physical functioning; RF, role functioning; EF, emotional functioning; CF, cognitive functioning; SF, social functioning, n (%) based on total sample (N=458); mean (SD) of QoL scales based on participants with valid QoL data (N=456).* *Scores transformed to 0–100 per EORTC QLQ-C30 manual. Higher scores = better QoL for functional/global health; higher scores = worse symptoms for symptom scales.*

**Table 2: EORTC QLQ-C30 symptom scores by socio-demographic and clinical characteristics among breast cancer patients**

| Characteristic | Fatigue | Nausea and Vomiting | Pain | Dyspnoea | Insomnia | Appetite loss | Constipation | Diarrhoea | Financial difficulties |
| --- | --- | --- | --- | --- | --- | --- | --- | --- | --- |
| Mean (SD) | 26.1 (21.4) | 6.4 (14.2) | 19.2 (24.8) | 7.9 (18.8) | 9.9 (20.7) | 15.1 (24.5) | 6.2 (16.5) | 1.9 (8.0) | 43.9 (33.7) |
| Age (Years) |  |  |  |  |  |  |  |  |  |
| <40 | 26.5 (21.0) | 1.99 (0.15) | 21.1 (24.6) | 7.6 (17.8) | 10.0 (18.8) | 15.6 (25.0) | 4.4 (11.9) | 1.8 (7.5) | 49.7 (33.9) |
| 40–59 | 24.5 (21.2) | 1.99 (0.13) | 16.5 (23.8) | 6.2 (16.8) | 8.2 (19.4) | 12.9 (22.6) | 6.5 (17.0) | 2.0 (8.5) | 40.3 (32.3) |
| ≥60 | 31.9 (23.1) | 2.00 (0.00) | 24.8 (28.4) | 16.4 (26.7) | 17.0 (29.0) | 23.3 (28.9) | 10.7 (24.3) | 1.9 (7.8) | 41.5 (36.9) |
| p | 0.077 | 0.848 | 0.025 | 0.002 | 0.066 | 0.023 | 0.382 | 0.997 | 0.016 |
| Residence |  |  |  |  |  |  |  |  |  |
| Urban | 25.3 (20.5) | 1.99 (0.11) | 17.8 (24.1) | 6.2 (16.2) | 8.4 (19.4) | 12.0 (22.2) | 5.2 (14.8) | 1.8 (7.9) | 37.5 (31.9) |
| Rural | 28.4 (23.8) | 1.98 (0.18) | 23.0 (26.3) | 12.7 (24.2) | 14.1 (23.6) | 23.7 (28.3) | 9.0 (20.3) | 2.3 (8.4) | 62.1 (32.0) |
| p | 0.295 | 0.438 | 0.033 | 0.003 | 0.003 | <0.001 | 0.052 | 0.478 | <0.001 |
| Study site |  |  |  |  |  |  |  |  |  |
| Black Lion | 28.9 (20.1) | 1.99 (0.13) | 20.6 (25.0) | 7.2 (17.4) | 7.8 (19.7) | 11.7 (21.8) | 4.4 (12.8) | 2.0 (8.5) | 37.5 (29.8) |
| Hiwot Fana | 36.1 (24.9) | 2.00 (0.00) | 30.3 (27.6) | 21.5 (28.5) | 25.1 (26.4) | 33.8 (28.6) | 11.3 (20.6) | 3.6 (10.4) | 59.0 (36.7) |
| Jimma | 16.5 (18.3) | 1.99 (0.17) | 11.4 (20.4) | 2.9 (10.9) | 6.4 (15.9) | 12.4 (23.1) | 7.1 (19.5) | 1.0 (5.6) | 48.3 (36.1) |
| p | <0.001 | 0.766 | <0.001 | <0.001 | <0.001 | <0.001 | 0.005 | 0.069 | <0.001 |
| Marital status |  |  |  |  |  |  |  |  |  |
| Married | 25.1 (21.3) | 1.99 (0.17) | 18.0 (24.7) | 7.1 (18.8) | 7.9 (19.2) | 14.5 (24.3) | 6.3 (16.8) | 0.9 (6.2) | 41.1 (34.4) |
| Unmarried | 27.7 (21.6) | 2.00 (0.00) | 21.1 (24.9) | 9.2 (18.8) | 13.0 (22.6) | 15.9 (24.8) | 6.1 (16.1) | 3.4 (10.2) | 48.3 (32.2) |
| p | 0.151 | 0.268 | 0.131 | 0.074 | 0.003 | 0.490 | 0.970 | <0.001 | 0.025 |
| Occupational status |  |  |  |  |  |  |  |  |  |
| Employed | 23.0 (20.2) | 2.00 (0.00) | 17.1 (23.0) | 5.3 (14.4) | 7.7 (17.3) | 10.8 (21.3) | 4.2 (13.2) | 2.1 (8.1) | 36.8 (32.5) |
| Unemployed | 29.1 (22.1) | 1.98 (0.18) | 21.1 (26.3) | 10.4 (21.9) | 12.0 (23.3) | 19.1 (26.5) | 8.1 (18.9) | 1.7 (8.0) | 50.6 (33.4) |
| p | 0.003 | 0.170 | 0.132 | 0.010 | 0.062 | <0.001 | 0.018 | 0.460 | <0.001 |
| Educational attainment |  |  |  |  |  |  |  |  |  |
| No formal education | 31.8 (23.9) | 2.00 (0.00) | 25.9 (28.0) | 13.4 (23.4) | 14.5 (25.6) | 21.7 (28.0) | 8.1 (18.4) | 2.6 (9.8) | 53.3 (34.6) |
| Primary & secondary | 24.2 (20.2) | 1.98 (0.20) | 15.9 (23.4) | 5.3 (15.4) | 7.8 (17.5) | 12.5 (22.8) | 6.0 (16.5) | 1.5 (6.8) | 43.4 (32.5) |
| Higher education | 21.3 (17.9) | 2.00 (0.00) | 15.5 (20.2) | 4.8 (15.1) | 7.1 (16.8) | 10.2 (19.4) | 3.7 (12.6) | 1.7 (7.4) | 30.3 (29.9) |
| p | <0.001 | 0.301 | <0.001 | <0.001 | 0.019 | <0.001 | 0.110 | 0.484 | <0.001 |
| Monthly household income (ETB) |  |  |  |  |  |  |  |  |  |
| ≤2,100 | 31.0 (21.9) | 1.99 (0.15) | 26.1 (26.5) | 11.6 (21.1) | 13.3 (22.8) | 19.9 (26.5) | 8.5 (18.8) | 3.2 (9.9) | 54.7 (30.1) |
| 2,101–3,254 | 24.9 (21.3) | 1.98 (0.18) | 17.1 (23.4) | 7.0 (18.1) | 8.9 (20.3) | 15.1 (24.7) | 6.5 (16.8) | 1.0 (7.2) | 47.7 (34.2) |
| 3,255–15,000 | 21.4 (19.9) | 2.00 (0.00) | 12.8 (21.8) | 4.4 (15.7) | 6.8 (17.7) | 9.4 (20.5) | 3.3 (12.6) | 1.1 (6.0) | 28.1 (31.4) |
| p | <0.001 | 0.584 | <0.001 | <0.001 | 0.009 | <0.001 | 0.005 | 0.008 | <0.001 |
| Distance to the health facility (km) |  |  |  |  |  |  |  |  |  |
| <5 | 25.4 (21.5) | 1.99 (0.11) | 19.6 (24.8) | 8.5 (19.3) | 10.1 (20.4) | 15.4 (24.4) | 6.7 (16.8) | 2.3 (8.8) | 42.7 (33.6) |
| ≥5 | 28.3 (21.0) | 1.98 (0.19) | 17.6 (25.0) | 5.9 (17.0) | 9.0 (21.8) | 14.0 (24.7) | 4.7 (15.5) | 0.6 (4.5) | 47.7 (33.7) |
| p | 0.150 | 0.378 | 0.464 | 0.165 | 0.299 | 0.455 | 0.118 | 0.060 | 0.165 |
| Health insurance |  |  |  |  |  |  |  |  |  |
| Yes | 26.2 (21.4) | 1.99 (0.15) | 18.4 (24.9) | 7.8 (18.6) | 9.9 (21.0) | 14.8 (24.6) | 6.3 (16.6) | 2.0 (7.9) | 43.7 (33.8) |
| No | 25.6 (21.8) | 2.00 (0.00) | 22.3 (24.5) | 8.4 (19.6) | 9.9 (19.6) | 16.1 (24.0) | 5.9 (16.2) | 1.5 (8.5) | 44.3 (33.4) |
| p | 0.803 | 0.481 | 0.085 | 0.759 | 0.798 | 0.457 | 0.949 | 0.318 | 0.862 |
| Total Treatment Interval (TTI) |  |  |  |  |  |  |  |  |  |
| ≤90 days | 20.3 (20.4) | 2.00 (0.00) | 13.0 (22.5) | 4.3 (16.1) | 7.0 (18.8) | 10.8 (24.5) | 5.5 (16.0) | 2.3 (9.4) | 35.8 (32.5) |
| >90 days | 28.5 (21.4) | 1.99 (0.16) | 21.7 (25.3) | 9.4 (19.6) | 11.0 (21.3) | 16.8 (24.3) | 6.5 (16.7) | 1.8 (7.5) | 47.2 (33.7) |
| p | <0.001 | 0.365 | <0.001 | <0.001 | 0.025 | 0.001 | 0.414 | 0.736 | 0.001 |
| Family size |  |  |  |  |  |  |  |  |  |
| <2 | 26.3 (20.1) | 2.00 (0.00) | 18.9 (24.1) | 7.6 (17.8) | 9.7 (21.6) | 12.8 (23.9) | 8.0 (18.6) | 2.4 (8.7) | 44.8 (33.8) |
| 2–5 | 25.6 (20.8) | 1.99 (0.16) | 18.3 (24.0) | 6.6 (17.0) | 8.8 (19.4) | 14.1 (23.3) | 5.5 (15.2) | 1.7 (7.8) | 42.0 (31.9) |
| >6 | 27.9 (25.8) | 2.00 (0.00) | 23.3 (28.8) | 14.4 (25.7) | 14.9 (24.3) | 22.6 (28.9) | 6.7 (18.8) | 2.1 (8.1) | 50.8 (40.4) |
| p | 0.858 | 0.582 | 0.555 | 0.016 | 0.086 | 0.031 | 0.520 | 0.622 | 0.223 |
| Stage at diagnosis |  |  |  |  |  |  |  |  |  |
| Stage I | 28.1 (14.2) | 2.00 (0.00) | 16.7 (16.7) | 3.9 (11.1) | 3.9 (11.1) | 15.7 (17.1) | 7.8 (18.7) | 0.0 (0.0) | 29.4 (26.0) |
| Stage II | 23.9 (21.0) | 1.99 (0.15) | 15.4 (23.0) | 5.7 (15.4) | 8.4 (19.5) | 12.1 (22.8) | 4.7 (13.7) | 1.6 (7.9) | 36.3 (31.1) |
| Stage III | 23.7 (21.6) | 1.99 (0.14) | 17.7 (24.7) | 7.6 (18.8) | 9.2 (19.8) | 13.0 (23.7) | 7.3 (18.4) | 2.4 (8.6) | 44.9 (33.5) |
| Stage IV | 40.9 (18.2) | 2.00 (0.00) | 36.5 (25.7) | 17.0 (26.1) | 18.7 (26.7) | 31.6 (27.8) | 6.4 (16.0) | 1.8 (7.5) | 67.3 (33.0) |
| p | <0.001 | 0.938 | <0.001 | 0.002 | 0.003 | <0.001 | 0.638 | 0.448 | <0.001 |
| Days since treatment initiation |  |  |  |  |  |  |  |  |  |
| Just started | 42.9 (30.8) | 2.00 (0.00) | 48.8 (28.8) | 28.6 (36.6) | 23.8 (30.5) | 40.5 (37.4) | 11.9 (24.8) | 4.8 (12.1) | 81.0 (28.4) |
| Early/Mid-treatment | 34.0 (21.4) | 2.00 (0.00) | 29.4 (25.3) | 12.7 (22.8) | 14.3 (24.4) | 28.3 (29.5) | 5.4 (14.0) | 2.5 (10.0) | 61.9 (32.5) |
| Late treatment | 30.1 (22.0) | 2.00 (0.00) | 21.5 (28.1) | 7.4 (18.0) | 11.2 (22.6) | 13.6 (22.5) | 7.0 (17.0) | 3.5 (10.3) | 48.4 (30.1) |
| Post-treatment | 20.5 (18.8) | 1.98 (0.18) | 12.4 (20.0) | 4.9 (14.2) | 6.8 (16.7) | 8.6 (18.2) | 6.0 (16.7) | 0.9 (5.5) | 32.7 (30.5) |
| p | <0.001 | 0.653 | <0.001 | <0.001 | <0.001 | <0.001 | 0.734 | 0.019 | <0.001 |
| Medically confirmed chronic illness |  |  |  |  |  |  |  |  |  |
| No | 25.9 (21.2) | 1.99 (0.15) | 18.6 (24.8) | 7.4 (18.7) | 9.7 (20.4) | 14.7 (24.4) | 6.1 (16.4) | 1.5 (7.4) | 45.0 (33.5) |
| Yes | 26.9 (22.4) | 2.00 (0.00) | 21.5 (24.8) | 10.0 (19.1) | 10.7 (21.9) | 16.5 (24.8) | 6.9 (17.0) | 3.4 (10.2) | 39.1 (34.2) |
| p | 0.810 | 0.493 | 0.256 | 0.090 | 0.747 | 0.476 | 0.592 | 0.028 | 0.134 |
| Treatment modality |  |  |  |  |  |  |  |  |  |
| Single modality | 36.7 (24.2) | 2 (0.00) | 35.8 (26.8) | 18.2 (27.6) | 17.8 (25.9) | 30.2 (30.1) | 6.7 (17.3) | 3.1 (9.8) | 66.7 (32.4) |
| Two modalities | 26.4 (19.7) | 2 (0.00) | 17.7 (23.8) | 6.7 (16.8) | 9.3 (20.3) | 14.0 (23.2) | 6.7 (16.8) | 2.0 (8.4) | 42.2 (31.8) |
| All three modalities | 15.1 (19.6) | 2 (0.00) | 9.3 (18.6 | 2.9 (10.8) | 4.5 (13.7) | 4.9 (15.0) | 4.1 (14.3) | 0.4 (3.7) | 28.8 (31.1) |
| p | <0.001 | 1.000 | <0.001 | <0.001 | <0.001 | <0.001 | 0.300 | 0.084 | <0.001 |
| Hospitalizations (past year) |  |  |  |  |  |  |  |  |  |
| 0–3 | 25.3 (20.9) | 1.99 (0.13) | 18.2 (24.1) | 6.9 (17.6) | 8.9 (19.3) | 14.1 (23.9) | 5.7 (15.9) | 1.4 (6.8) | 43.8 (33.7) |
| 4–7 | 48.6 (24.3) | 2.00 (0.00) | 45.8 (28.9) | 35.4 (28.5) | 37.5 (34.2) | 41.7 (25.8) | 20.8 (24.0) | 14.6 (21.0) | 45.8 (34.2) |
| p | <0.001 | 0.788 | <0.001 | <0.001 | <0.001 | <0.001 | <0.001 | <0.001 | 0.869 |
| BMI (kg/m²) |  |  |  |  |  |  |  |  |  |
| Underweight (<18.5) | 38.7 (26.1) | 2.00 (0.00) | 28.7 (33.5) | 14.7 (25.6) | 22.7 (28.4) | 33.3 (31.9) | 10.7 (20.9) | 4.0 (11.1) | 61.3 (39.3) |
| Normal (18.5–24.9) | 26.2 (20.2) | 1.99 (0.15) | 18.1 (23.6) | 7.1 (17.7) | 9.1 (20.1) | 14.5 (23.7) | 5.0 (14.0) | 1.9 (8.2) | 43.5 (32.1) |
| Overweight (25–29.9) | 27.3 (23.8) | 2.00 (0.00) | 24.5 (27.8) | 11.6 (22.5) | 11.1 (21.7) | 15.7 (25.6) | 8.8 (21.7) | 1.4 (6.7) | 45.8 (34.7) |
| Obese (30–39.9) | 9.7 (15.1) | 2.00 (0.00) | 9.0 (15.5) | 1.4 (6.8) | 4.2 (11.3) | 2.8 (9.4) | 11.1 (23.4) | 1.4 (6.8) | 25.0 (37.1) |
| p | <0.001 | 0.868 | 0.032 | 0.019 | 0.006 | <0.001 | 0.216 | 0.504 | 0.003 |
